# The Interplay Between Multisensory Processing and Attention in Working Memory: Behavioral and Neural Indices of Audiovisual Object Storage

**DOI:** 10.1111/psyp.70018

**Published:** 2025-02-21

**Authors:** Ceren Arslan, Daniel Schneider, Stephan Getzmann, Edmund Wascher, Laura‐Isabelle Klatt

**Affiliations:** ^1^ Leibniz Research Centre for Working Environment and Human Factors Dortmund Germany

**Keywords:** alpha oscillations, EEG, multisensory processing, selective attention, working memory

## Abstract

Although real‐life events are multisensory, how audio‐visual objects are stored in working memory is an open question. At a perceptual level, evidence shows that both top‐down and bottom‐up attentional processes can play a role in multisensory interactions. To understand how attention and multisensory processes interact in working memory, we designed an audiovisual delayed match‐to‐sample task in which participants were presented with one or two audiovisual memory items, followed by an audiovisual probe. In different blocks, participants were instructed to either (a) attend to the auditory features, (b) attend to the visual features, or (c) attend to both auditory and visual features. Participants were instructed to indicate whether the task‐relevant features of the probe matched one of the task‐relevant feature(s) or objects in working memory. Behavioral results showed interference from task‐irrelevant features, suggesting bottom‐up integration of audiovisual features and their automatic encoding into working memory, irrespective of task relevance. Yet, event‐related potential analyses revealed no evidence for active maintenance of these task‐irrelevant features, while they clearly taxed greater attentional resources during recall. Notably, alpha oscillatory activity revealed that linking information between auditory and visual modalities required more attentional demands at retrieval. Overall, these results offer critical insights into how and at which processing stage multisensory interactions occur in working memory.

## Impact statement

Our findings offer prominent insights into multisensory working memory. When confronted with concurrent auditory and visual features, information from both modalities is combined into a working memory representation, irrespective of task relevance. Notably, when the task instructions explicitly involve the storage of audiovisual objects as a whole, recall requires more attentional resources, as indexed by alpha power modulations.

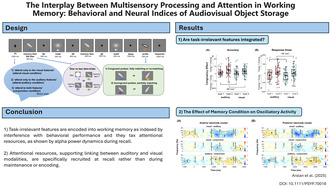

## 1 Introduction

To navigate through a complex, multisensory environment, our senses need to interact with each other constantly. This applies not only to low‐level perceptual mechanisms but also to higher‐order cognitive functions such as working memory. However, current working memory research is strongly dominated by vision, and our understanding of how multisensory objects are encoded and maintained in working memory remains limited. The current study sets out to investigate how audiovisual information is stored in working memory and aims to observe neural correlates of attentional mechanisms supporting the linking of cross‐modal information.

The concurrent storage of auditory and visual items has been predominantly investigated in a line of behavioral studies, adopting a dual‐task paradigm (Cowan et al. 2014; Fougnie et al. 2015; Morey and Cowan 2004, 2005; Saults and Cowan 2007). Such studies primarily address the question as to whether storage limits in working memory are restricted by a central, amodal capacity or governed by domain‐independent storage systems—a question that remains controversially debated and is beyond the scope of this article. Notably, these studies aim to isolate the subcomponents of working memory, as proposed by the classical modular view (Baddeley 1986), rather than investigating multisensory working memory representations. Specifically, in most studies, the unimodal memory arrays are presented in spatiotemporal asynchrony, or only one of the modalities is probed during recall. Consequentially, previous studies did not create an incentive to encode or store cross‐modally presented features as integrated audiovisual objects.

The question of how audiovisual information is encoded and maintained in working memory ultimately also taps into the debate on whether individual features are stored separately or integrated as a bound object representation and to what extent the latter include task‐irrelevant features. With respect to the perceptual aspects of cross‐modal binding, we can draw on an ample body of studies in the multisensory processing literature, showing that the combination of information from different sensory modalities into a single multisensory event or representation (i.e., multisensory integration) can occur in a purely bottom‐up manner if certain conditions are met, but requires an additional top‐down attention mechanism otherwise (Busse et al. 2005; Matusz et al. 2019; Sarmiento et al. 2016; Senkowski et al. 2005, 2007, 2009). According to a framework by Talsma et al. (2010), the interplay between attention and multisensory processing is determined by the physical complexity of the environment. Cross‐modal interactions can happen in a bottom‐up fashion if the competition for a limited capacity between stimuli within a given modality is low. Accordingly, the co‐occurrence of auditory and visual stimuli in space and time will likely promote the cross‐modal spread of attention toward task‐irrelevant features of an attended object. Supporting this idea, it has been shown that a task‐irrelevant and spatially uninformative sound that is presented concurrently with a change of a visual target item facilitates visual search task performance (Van Der Burg et al. 2008, 2010). This observation has been interpreted as evidence for the bottom‐up driven integration of the task‐irrelevant sound and the visual target, making the visual target pop out from the cluttered scene.

Critically, whether incidentally or intentionally formed cross‐modal feature bindings are also maintained in an integrated manner in working memory or whether the task‐irrelevant feature is dropped from working memory remains unclear. For visual working memory, a growing body of evidence suggests that object representations retain both a feature‐ and an object‐level (Brady et al. 2011; Li et al. 2022). However, findings concerning the storage of task‐irrelevant features remain somewhat contradictory. While some authors were able to successfully decode task‐irrelevant features based on multivariate electroencephalography data (Chen et al. 2021), others were only able to decode task‐relevant features (Bocincova and Johnson 2019; Yu and Shim 2017). On the contrary, behavioral findings, such as intertrial priming (Jiang et al. 2016) and attentional capture in a secondary search task (Harrison et al. 2021), corroborate the idea that at least some residual representation of task‐irrelevant visual features is retained. Likewise, for auditory working memory, there is behavioral evidence showing better recall performance when participants are required to recall an auditory object as a whole compared to when they are asked to report individual features of the object, suggesting that auditory features might be naturally stored as bound objects (Joseph et al. 2015). Accordingly, Bays et al. (2022) postulated that task‐irrelevant features of attended objects are automatically encoded, but not actively or only weakly maintained through the engagement of top‐down resources. Yet, to date, with respect to multisensory object representations, this question remains largely unaddressed.

Although the representational format of multisensory information in working memory is not clear to this date, multiple working memory studies combining modality‐specific or cross‐modal stimulus presentation with electroencephalography (EEG), or magnetoencephalography have illuminated the role of oscillatory mechanisms from various angles. Specifically, three major lines of research can be identified: One area of investigation has focused on identifying modality‐independent, supramodal substrates of working memory retention or working memory control (Cowan et al. 2011; Majerus et al. 2016; Spitzer and Blankenburg 2012; van Ede et al. 2017). In addition, several studies suggest that functional connectivity dynamics, supporting long‐range communication between distant brain areas, may play an important role in the encoding of audiovisual objects (Xie et al. 2021), cross‐modal integration (van Driel et al. 2014), as well as cognitive control of audiovisual working memory maintenance (Daume et al. 2017). Finally, a large body of literature delineates the key role of alpha (8–13 Hz) power modulations in facilitating unisensory (Dubé et al. 2013; Payne et al. 2013) as well as cross‐modal (Foxe et al. 1998; Mazaheri et al. 2014) information processing by gating information between sensory regions (for review see Jensen and Mazaheri 2010). Extending this work from the perceptual domain, several working memory studies have demonstrated that alpha power likewise increases over mnemonically irrelevant sensory regions, while decreasing over task‐relevant regions (Spitzer and Blankenburg 2012; van Ede et al. 2017), corroborating its functional significance in the dynamic (dis‐)engagement and functional inhibition of sensory areas.

Here, we recorded the EEG while participants completed three variants of an audiovisual delayed match‐to‐sample task. In two single‐feature conditions, participants were instructed to attend to either the visual or the auditory features of the presented audiovisual objects, while in the conjunction condition (i.e., audiovisual condition), participants attended to both features. Compared to previous behavioral and EEG studies, the present design entails several key advances. Foremost, by always presenting an audiovisual (rather than a unisensory) probe, we prevent that the task itself entails an incentive to store auditory and visual features in a segregated manner. Further, by presenting the auditory and visual features at the same time and location, we promote the bottom‐up integration of cross‐modal input and thereby enhance the perceptual impression that both features originate from the same source and belong to the same multisensory object. Finally, we introduce a novel conjunction condition, in which participants are explicitly instructed to consider the auditory and visual features as part of the same object. Thereby, we create three variants of the task that are identical in terms of their physical stimulation (i.e., always present audiovisual input) but differ in terms of their task instruction.

The present study aims to address three major questions: First, by assessing behavioral interference effects of the task‐irrelevant features in the single‐feature conditions, we aim to explore to what extend task‐irrelevant cross‐modal features are maintained in working memory. If the task‐irrelevant features of memory items are stored to some degree, then they should interfere with the task performance; decreasing the accuracy and/or increasing reaction times (RTs) when their comparison with the probe would imply a different response than the task‐relevant feature. In line with the framework by Talsma et al. (2010), we expect the extent of interference to be modulated by memory load, such that under low working memory load (i.e., in one‐item trials), both task‐relevant and task‐irrelevant features of a multisensory object are maintained (i.e., larger interference effects), whereas under high‐working memory load, fewer resources are devoted to the maintenance of task‐irrelevant features, resulting in their more rapid decay or removal (i.e., smaller interference effects). In addition, by exploiting well‐established event‐related potentials (ERPs) of auditory and visual working memory storage, we aim to track whether those task‐irrelevant features are actively maintained. Such that if task‐irrelevant auditory features are maintained in the visual condition, sustained anterior negativity (SAN) amplitudes (i.e., an ERP correlate for auditory working memory storage, Alunni‐Menichini et al. 2014; Guimond et al. 2011; Lefebvre et al. 2013; Nolden et al. 2013) should vary as the number of auditory features increases. Similarly, if visual features are actively maintained in the auditory condition, an analogous load effect should be visible in posterior negative slow‐wave amplitudes (i.e., negative slow wave (NSW), an ERP correlate for visual working memory storage, Diaz et al. 2021; Feldmann‐Wüstefeld 2021) as visual features increase in number. Second, by contrasting the behavioral set size effect across the three conditions, we aim to disentangle whether audiovisual features are stored in a feature‐based or object‐based fashion. Third, while previous EEG studies have primarily focused on revealing the neurocognitive mechanisms supporting the prioritization of one modality over the other, the present study illuminates the oscillatory mechanisms involved in linking information across modalities by contrasting the single‐feature conditions with the novel cross‐modal conjunction condition. By inspecting alpha power modulations, the current study aims to observe the (re‐)distribution of attentional resources when unimodal and cross‐modal information is encoded, maintained, and retrieved.

## 2 Materials and Method

### 2.1 Ethics Statement

This study was approved by the Ethical Committee at the Leibniz Research Centre for Working Environment and Human Factors. It was carried out in line with the guidelines of the Declaration of Helsinki. All participants provided written informed consent before the experimental session. We compensated the participants with either 12 euros per hour or, if requested, course credits.

### 2.2 Participants

Fifty participants took part in the study. Two participants were excluded from the dataset due to a disconnected ground electrode during EEG recording. Three participants were discarded from the dataset due to behavioral performance below or close to the chance level (19%, 34%, and 56%, respectively). Finally, one participant was excluded due to a lack of cooperation in following the task instructions. The final sample included 44 participants (15 men, 29 women, and 3 left‐handed) with a mean age of 23.18 years (SD = 3.59, age range = 18–34). This corresponds to the targeted sample size that was determined using an a priori power analysis (see “Power analysis” section). All participants reported no history of neurological disorders or psychopharmacological medication.

We tested the hearing acuity of each participant using a pure‐tone audiometry (Oscilla USB 330; Inmedico, Lystrup, Denmark), presenting eleven pure‐tone frequencies in between 125 and 8000 Hz. All participants showed normal hearing levels below or equal to 25 dB for frequencies between 125 Hz and 4 kHz. For frequencies higher than 4 kHz (i.e., 6 and 8 kHz), five participants displayed marginally elevated hearing levels of 30 dB (3 participants at 6 kHz and 1 participant at 8 kHz) and 35 dB (1 participant at 6 kHz). Considering that all experimental auditory stimuli presented with a frequency below 4 kHz, these outliers were negligible, so participants were not discarded from the dataset.

Furthermore, we tested each participant's visual acuity using Landolt C optotypes at a 1.5 m distance. The average visual acuity in the sample was 1.62 (SD = 0.32, range = 1.2–2.6), reflecting sufficient vision. Since arithmetically averaged visual acuity values can be flawed, we applied a logarithmic averaging procedure to obtain the mean (Bach and Kommerell 1998). That is, the logarithm of the individual visual acuity measures was obtained before averaging. Then the antilogarithm of the subsequent average value was computed.

### 2.3 Power Analysis

Given a lack of suitable EEG studies, the sample size rationale emphasized maximizing power for the behavioral effects of interest. To determine the smallest effect size of interest (Lakens 2022) effect sizes from previous studies with similar research aims and experimental designs were considered. Our review revealed that effect sizes of earlier studies ranged between 0.16 and 0.84 𝜂^2^_*p*_, the former effect being related to an interaction between probe congruency and memory conditions (single modality vs. conjunction conditions) on response times (Daume et al. 2017)^1^. Thus, we determined our smallest effect size of interest to be 0.16 𝜂^2^_*p*_. For an overview of the studies that informed this choice, please see the associated pre‐registration at https://osf.io/q6wzr. Power analysis using MorePower 6.0 (Campbell and Thompson 2012) targeting an effect size of 0.16 𝜂^2^_*p*_, a power of 80%, and an alpha level of 0.05 for a 2 × 2 interaction resulted in a sample size estimate of 44 participants.

### 2.4 Experimental Setup and Stimuli

The experiment was conducted in a dimly lit, sound‐attenuated room (5.0 × 3.3 × 2.4 m^3^). The background noise level was kept below 20 dB(A) using foam panels on the walls and ceiling and a woolen carpet on the floor. Stimulus presentation was governed using the E‐Prime 3.0 software (Psychology Software Tools, Pittsburgh, PA). We used an AudioFile Stimulus Processor to control for the synchronization of auditory stimuli with EEG triggers (Cambridge Research Systems, Rochester, UK). Additionally, we tested the synchronization of auditory and visual stimuli. Visual onset timing was assessed using an analogue optical sensor, which was sensitive to the luminance change on the screen. To measure the onset of the auditory stimuli, the output of the AudioFile Stimulus Processor was fed into the EEG recording, using a NeurOne Isolation Box (Bittium Biosignals Ltd., Kuopio, Finland) for external analog signals. This allowed us to record the sound signal transmitted to the loudspeakers and, thus, quantify the onset of the sound wave. According to our measurements, the two signals were highly synchronous with a negligible difference of ~0.5 to 3 ms. Note that time measurements were obtained in a separate recording session, in which all combinations of visual and auditory stimuli were (dis)played.

Visual stimuli were displayed on a 49″ centrally aligned 1800R curved monitor with a 5120 by 1440‐pixel resolution and a 100 Hz refresh rate (Samsung, Seoul, South Korea). A full‐range loudspeaker (SC 55.9–8 Ohm; Visaton, Haan, Germany) was centrally mounted below the screen. Participants were seated in a comfortable chair at a distance of 130 cm from the screen.

As auditory stimuli, eight pure tones with frequencies ranging from 270 to 3054 Hz (in steps of half an octave: 270, 381, 540, 763, 1080, 1527, 2159, and 3054 Hz) were generated using the MATLAB (R2022a) function “pure tone generator” (Wojcicki 2024). Tones had 20 ms fade‐in and fade‐out time windows with a sampling frequency of 44,100 Hz and in full scale (amplitude = 1). A normalized auditory mask stimulus was generated by mixing all eight tones. As an auditory filler item, we created a white noise with an amplitude of 0.5 using free software called Audacity (version 3.2.0). On average, auditory stimuli were presented at a sound level of 76 dB (LAeq, A‐weighted, equivalent continuous sound level).

As visual stimuli, eight tear‐drop‐shaped orientations (RGB values 208, 208, 208) with angles between 22.5° and 337.5° (22.5°, 67.5°, 112.5°, 157.5°, 202.5°, 247.5°, 292.5°, and 337.5°) were generated using the MATLAB (R2022a) function “polarplot.” The size of the tear‐drop stimuli (from the tip to the opposite end) was 2°. A visual mask stimulus was generated by overlaying all eight orientations. Finally, as a visual filler item, we created a white‐blurred circle (RGB values 255, 255, 255 with a visual angle of 2.3°) using the MATLAB (R2022a) function “polarplot.” All stimuli were presented in front of a light gray background (RGB values 128, 128, 128).

### 2.5 Procedure, Task, and Experimental Design

2.5.1

**FIGURE 1:**
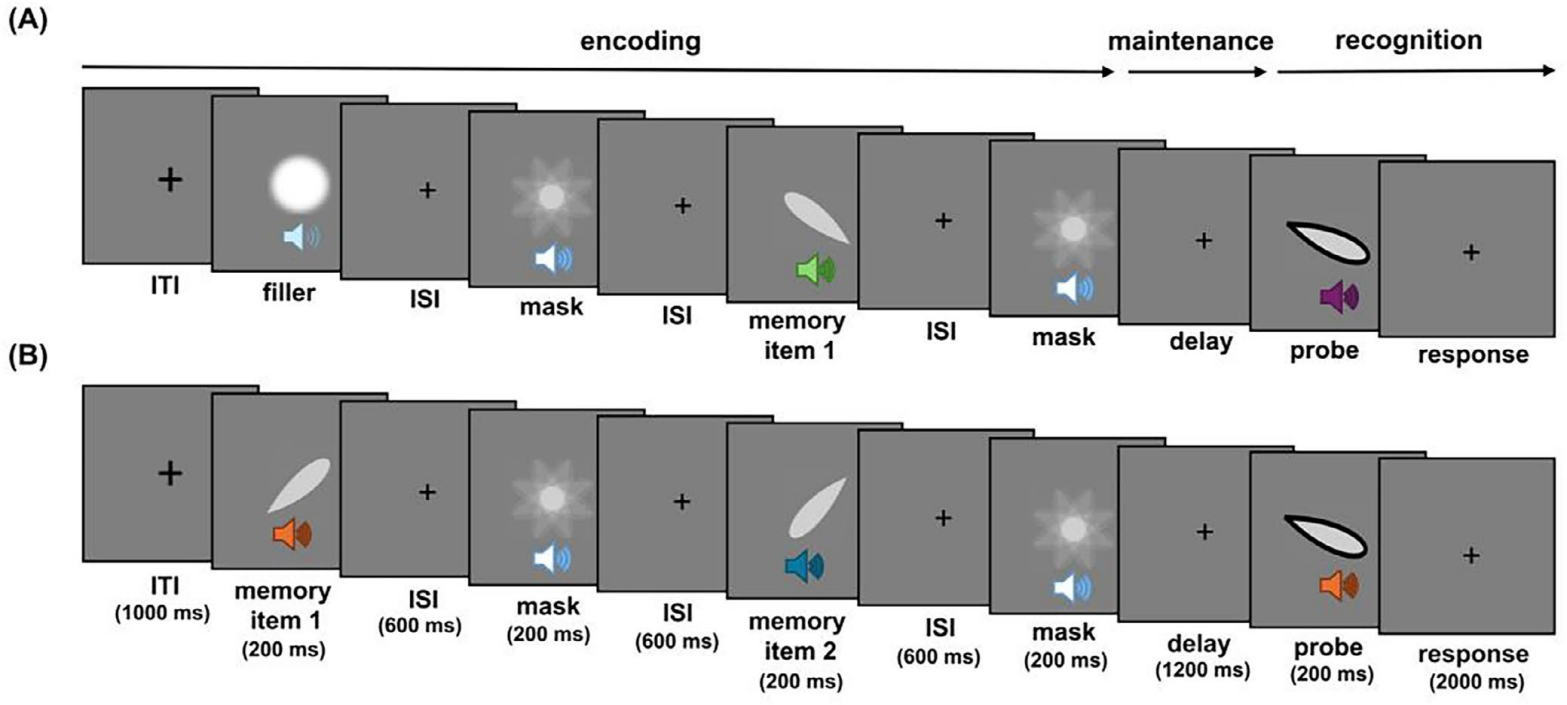
Schematic illustration of one versus two‐item trials. (A) In one‐item trials, the first item was an audiovisual filler item; (B) while in two‐item trials, this was replaced by a memory item. The three memory conditions only differed in terms of their task instructions, while the physical stimulation was always the same. ISI, inter‐stimulus interval; ITI, inter‐trial interval.

Participants performed an audiovisual delayed match‐to‐sample task. The sequence of events within a trial is illustrated in Figure 1. A briefly flashing fixation cross (100 ms) signaled the start of each trial 1000 ms before the beginning of the trial sequence. Then, participants were presented with one or two sequentially displayed audiovisual memory items: spatially and temporally aligned tones, varying in frequency, and teardrops, varying in orientation. The tone‐teardrop pairs were randomly chosen on each trial. All memory items were presented in a central location (0° azimuth angle) for 200 ms. To minimize the impact of sensory memory traces, each memory item was followed by a centrally presented audiovisual mask for 200 ms. Memory items and masks were separated by an interstimulus interval of 600 ms. Trials with one or two memory items were randomly intermixed. In one‐item trials, an audio‐visual filler stimulus replaced the first memory item to equate the overall trial length. Finally, following a delay period of 1200 ms, an audiovisual probe stimulus was displayed for 200 ms, and participants were asked to indicate whether the task‐relevant probe features matched one of the items currently held in memory. Responses were recorded within a 2200 ms response period (relative to the onset of the probe) using a response pad with two horizontally arranged buttons. Each participant made button presses using left and right thumbs to respond. The assignment of response keys (left vs. right) to response options (“yes” vs. “no”) was counterbalanced across participants. The speed and accuracy of the responses were equally emphasized. Again, 2000 ms after probe offset, a brief flashing of the fixation cross (100 ms) signaled the start of the next trial.

Overall, the experiment consisted of three blocked memory conditions: in the auditory condition, participants were instructed to attend only to the auditory features, while in the visual condition, they focused only on the visual features. In the following, the auditory and visual conditions are also referred to as the single‐feature conditions. In the conjunction condition, participants were asked to attend to both the auditory and visual features and compare the whole object to the audiovisual probe features. Critically, participants were explicitly instructed in conjunction blocks to consider both features as part of the same object. It is important to note that the structure of the stimulus sequence remained identical in all three conditions, such that participants were always presented with audiovisual input. That is, the three memory conditions only differed in terms of their task instructions.

**FIGURE 2:**
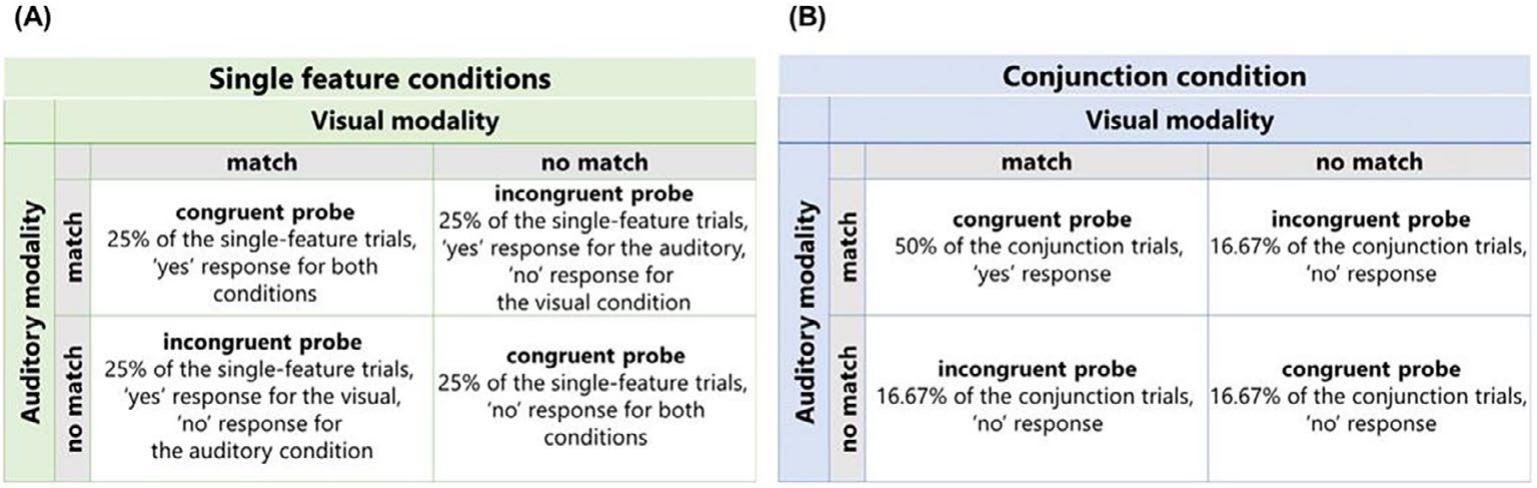
Illustration of probe types and respective proportion of trials per memory condition. (A) In single‐feature conditions, congruent and incongruent probe types appeared equally often; (B) to equalize “yes” and “no” responses across probe types in the conjunction condition, the “auditory match + visual match” probe type was presented in half of the conjunction condition trials, while the other three probe conditions were equally distributed across the remaining 50% of trials.

In addition to memory condition and set size, we also manipulated the type of probes. When combining stimulus features from two modalities, there are four resulting types of probes, depending on which features of the probes match or do not match the memory items. Essentially, those four probe types break down into two categories: (a) congruent probes, for which features in both modalities indicate a match or a no‐match (i.e., auditory feature match + visual feature match; auditory feature no‐match + visual feature no‐match) and (b) incongruent probes, for which features in one modality indicate a match while the other modality indicates a no‐match (i.e., auditory feature no‐match + visual feature match; auditory feature match + visual feature no‐match). Each probe type appeared equally often in the single‐feature conditions (i.e., auditory and visual memory conditions) (see Figure 2A). This also results in an equal number of “yes” and “no” responses. However, considering both features were task‐relevant in the conjunction condition, the proportion of trials per probe type had to be adjusted to balance the number of “yes” and “no” responses. Congruent‐match trials (i.e., “auditory feature match + visual feature match” probe) requiring a “yes” response constituted 50% of trials in the conjunction condition. The other three probe types, which required a “no” response in the conjunction condition, appeared equally often in one‐third each of the remaining 50% of trials (see Figure 2B). Across all memory conditions, the nonmatching feature of the probe was always a novel stimulus, different from the features presented in each trial, randomly drawn from the remaining pool of unimodal features.

Each memory condition included 240 trials (i.e., 120 one‐item trials and 120 two‐item trials), resulting in 720 trials. The experimental session was divided into 15 mini‐blocks (five mini‐blocks for each memory condition) of 48 trials. Each mini‐block lasted approximately five minutes. The order of the mini‐blocks was pseudorandomized such that memory conditions for each set of three blocks were randomly drawn from all three possible memory conditions until all conditions were chosen. The same procedure was followed for the next set of three mini‐blocks, except that the first block of the next set could not have the same memory condition as the last block of the previous set had. Following each mini‐block, participants were allowed to take a self‐paced break. Preceding the start of the experimental session, participants practiced the task by performing 24 trials for each memory condition. After the experimental task, participants reported their ability to attend only to the task‐relevant features and to ignore the task‐irrelevant features in a follow‐up questionnaire, which is included in the Supporting Information (see Section 5). Overall, the entire task procedure (excluding EEG preparation time) took approximately one and a half hours.

### 2.6 EEG Recording

We used a 64 Ag/AgCl electrode cap (BrainCap; Brainvision, Gilching, Germany) with electrodes positioned according to the international 10–20 system (Pivik et al. 1993) to record EEG. The signal was sampled at 1000 Hz (NeurOne Tesla amplifier, Bittium Biosignals Ltd., Kuopio, Finland), and impedances were kept below 20 kΩ. The AFz and FCz electrodes served as ground and reference electrodes, respectively.

### 2.7 EEG Preprocessing

We used MATLAB (R2022a) and the open‐source toolbox EEGLAB (2022.1; Delorme and Makeig 2004) for preprocessing. The continuous data were filtered offline using a 0.01 high‐pass filter (filter order: 330001, transition bandwidth: 0.01 Hz, −6 dB cutoff: 0.005 Hz) and a 40 Hz low‐pass filter (filter order: 331, transition bandwidth: 10 Hz, −6 dB cutoff: 45 Hz). Channels with a normalized kurtosis surpassing five standard deviations of the mean were rejected through an automated channel rejection procedure in EEGLAB (i.e., pop_rejchan function). On average, 4.64 channels were rejected per participant (range = 0 to 9, SD = 1.82). Removed channels were interpolated using the spherical spline of the neighboring channels. Subsequently, the data were re‐referenced to the average of all channels.

Next, we employed a semiautomatic artifact rejection method based on a rank‐reduced independent component analysis (ICA). To this end, first, the continuous data were downsampled to 200 Hz to achieve a faster ICA decomposition. In addition, to increase the percentage of near‐dipolar components, data were filtered with a 1 Hz high‐pass filter (Winkler et al. 2015). Finally, to further reduce computation time, every other trial was selected after segmenting the data into epochs ranging from −1700 to 5500 ms relative to the onset of the first item. Finally, optimizing the signal‐to‐noise ratio in the ICA input, an automatic trial‐rejection method was implemented (i.e., function pop_autorej) to exclude trials containing major artifacts and large voltage fluctuations (> 1000 μV). This procedure rejects trials, including data surpassing a given standard deviation in an iterative process (threshold: 5 SD, maximum percentage of total rejection per iteration: 5%). Subsequently, ICA was computed.

The hereby obtained ICA decomposition was then copied to the original, filtered (0.01 Hz high‐pass filter), and re‐referenced continuous data with the initial sampling rate of 1 kHz. This procedure allows us to use an optimized high‐pass filter cutoff for ICA while keeping a different high‐pass filter cutoff that is more suitable for the planned ERP analysis. Again, the data were epoched from −1700 to 5500 ms relative to the onset of the first item and were baseline‐corrected with a prestimulus baseline period of −700 to 0 ms. To identify artefactual independent components (ICs) reflecting eye movements, heart‐ or muscle activity, line noise, channel noise, or other non‐brain activity, we used the EEGLab plugin ICLabel (v1.4; Pion‐Tonachini et al. 2019). ICs that were given a probability estimate below 50% for the brain category were rejected from the dataset. Consequentially, 30.68 components (SD = 7.19) were excluded per participant on average. The remaining trials with large fluctuations (i.e., exceeding threshold limits of −150 to 150 μV) were excluded using the EEGLAB function “pop_eegthresh.” On average, 226.77 trials (SD = 17, 94.83%) remained in the auditory, 230.25 trials (SD = 16.07, 95.94%) in the visual, and 229.82 trials (SD = 16.87, 95.77%) in the conjunction condition.

### 2.8 Time‐Frequency Decomposition

To obtain event‐related spectral perturbations (ERSPs; Makeig et al. 2004), Morlet wavelet convolution was applied using the EEGLAB routines calling the *pop_newtimef* function. Segmented EEG data were convolved with complex Morlet wavelets ranging in frequencies between 4 and 40 Hz (raising logarithmically in 52 steps). Aiming to obtain a good balance between temporal and frequency precision, the number of cycles increased by a factor of 0.5 with increasing frequency. At the lowest frequency, the number of cycles was 3, whereas at the highest frequency, it was 15.

The data were baseline‐corrected, using a common trial average prestimulus baseline (dividing by average power across trials at each frequency, i.e., “gain model”), ranging from −600 to −200 ms before the onset of the first item. Compared to a condition‐specific baseline correction, this procedure increases the signal‐to‐noise ratio in the baseline period. Before the common baseline procedure, a full‐epoch length single‐trial correction was applied (Grandchamp and Delorme 2011). The resulting ERSPs ranged from −1282 to 5082 ms.

### 2.9 Statistical Analyses

Statistical analyses were carried out using MATLAB (R2022a) and JASP (version 0. 16.4.0). Unless otherwise specified, the significance of all tests was assessed at an alpha level of 0.05. For analyses of variance, we report Greenhouse–Geisser corrected *p*‐values in case the assumption of sphericity was violated (Mauchley's test *p* < 0.05). For repeated measures ANOVA and paired‐sample *t*‐tests, we report partial eta squared (𝜂^2^_*p*_) and Cohen's *d* (equation 10 in Lakens 2013) as effect sizes, respectively. Bonferroni–Holm method was used to correct multiple comparisons, and corrected *p*‐values are described as *p*_corr_. Bayes Factors (BF_10_) were obtained as a supplementary measure using JASP. According to Quintana and Williams (2018), a BF_10_ greater than 3, 10, or 30 is considered as moderate, strong, or very strong evidence in favor of the alternative hypothesis, respectively. A BF_10_ larger than 100 suggests extreme positive evidence. Analogously, a BF_10_ smaller than 0.33, 0.1, or 0.03 is considered as moderate, strong, or very strong evidence in favor of the null hypothesis, respectively, while a BF_10_ smaller than 0.01 is indicative of extreme evidence for the null hypothesis. For cluster‐based permutation tests, Cohen's *d* is reported as the effect size measure (equation 10 in Lakens 2013). Here, we calculated effect sizes for significant clusters by using the average over cluster method (Meyer et al. 2021). All cluster‐based permutation tests were carried out using the MATLAB toolbox Fieldtrip (Version 20,230,422; Oostenveld et al. 2011).

#### 2.9.1 Behavioral Analyses

Mean RTs for correct trials and mean accuracy (percentage of correct responses) were calculated to assess participants’ behavioral performance in the audiovisual delayed match‐to‐sample task. Responses that occurred after 2000 ms following the offset of the probe or occurred prematurely (i.e., within 150 ms following the onset of the probe) were excluded from further analyses.

##### 2.9.1.1 Storage of Task‐Irrelevant Features

First, to investigate to what extent the task‐irrelevant feature of an attended object is still represented in working memory, we explored the effect of probe congruency in single‐feature conditions. The conjunction condition was excluded from these analyses, given that both probe dimensions were task‐relevant. To this end, accuracy and RT differences between trials with congruent probes (i.e., both probe‐features match one of the memory items or both probe‐features do not match one of the memory items) and incongruent probes (i.e., only one probe‐feature matches one of the features of memory items, whereas the other probe‐feature has not been previously presented in a given trial) were computed. These differences between congruent and incongruent probes served as the dependent variables for two 2 × 2 repeated‐measures analysis of variance (rmANOVA)s with set size (set size 1 vs. 2) and condition (auditory vs. visual) as within‐subject factors.

To observe whether the degree of interference from an incongruent probe depends on whether it is a task‐relevant or irrelevant feature, an additional analysis contrasted the effect of probe congruency between the single‐feature conditions and the conjunction condition. For this analysis, the differences in RT and accuracy between congruent and incongruent probe trials, which required only “no” responses, served as a dependent variable. The main findings are briefly reported, but for details, the reader is referred to the Supporting Information.

##### 2.9.1.2 Representational Format: Features Versus Objects

Second, to explore to what extent an expected set size effect (i.e., lower accuracy and increased RTs as the set size increases) is driven by the number of features or the number of objects, two rmANOVAs were performed, including the factors set size (set size 1 vs. 2) and memory condition (auditory, visual, and conjunction conditions) as within‐subject factors for accuracy and RT.

#### 2.9.2 ERP Analyses

The SAN and the posterior NSW were chosen as EEG correlates of auditory and visual working memory load, respectively. Based on previous work, mean SAN amplitudes were calculated in a cluster of frontocentral electrodes, including AF3/4, F3/4, FC3/4, Fz, and FCz (Alunni‐Menichini et al. 2014; Guimond et al. 2011). Mean NSW amplitudes were assessed in a cluster of parieto‐occipital electrodes, including channels P7/8, P3/4, Pz, PO7/8, PO3/4, O1/2, and Oz (Diaz et al. 2021). Mean amplitudes were calculated during the maintenance period, ranging from 100 ms post‐mask offset to probe onset (i.e., between 2700 and 3800 ms following the onset of the first item). The first 100 ms after mask offset were excluded to avoid including any sensory activity related to the sensory processing of the mask.

To observe if task‐irrelevant features were actively maintained in working memory regardless of the task instructions, set size effects (i.e., paired‐sample *t*‐tests contrasting set size 1 vs. 2) were assessed for (a) SAN amplitudes in the visual condition and (b) NSW amplitudes in the auditory conditions. Mean amplitudes were submitted to a rmANOVA, including the within‐subject factors: memory conditions (auditory vs. visual vs. conjunction) and set size (set size 1 vs. 2). Further, a series of pre‐registered, planned contrasts were conducted: To verify that the SAN and the NSW were sensitive to the set size manipulation of the task‐relevant modality, one‐sided paired sample *t*‐tests were carried out, contrasting mean amplitudes between one‐ and two‐item trials in each condition.

#### 2.9.3 Time‐Frequency Analysis

Cluster‐based permutation tests were conducted using the fieldtrip toolbox to identify frequency ranges and time windows sensitive to our experimental manipulations. Unless otherwise specified, default parameters were used (for details, see “Cluster‐based permutation tests” section). To reduce the dimensionality of the data, we focused the analysis on two electrode clusters including an anterior electrode cluster: C1/C2/C3/C4/Cz, FC1/FC2/FC3/FC4, F1/F2/F3/F4/Fz, and a posterior electrode cluster: PO3/PO4/PO7/PO8/PO9/PO10, P3/P4, O1/O2/O9/O10/Oz identified by a task‐independent localizer procedure, which is discussed in the Supporting Information (for details, see Section 2).

First, to test for the condition‐dependent time‐frequency modulations (irrespective of set size), the following pair‐wise contrasts were created: visual vs. auditory, visual vs. conjunction, and auditory vs. conjunction conditions. For both the anterior and posterior clusters, the time‐frequency data was averaged across the two types of set size. Then, cluster‐based permutation tests were run using all frequencies between 4 and 40 Hz and the entire epoch length, ranging from 1200 ms before and 5000 ms following the first item onset.

Second, the set size effect (set size 2 vs. 1) was tested separately for all three memory conditions. For both anterior and posterior clusters, time‐frequency data were submitted to a series of cluster‐based permutation tests, considering all frequencies (4– 40 Hz) and the entire epoch duration.

Third, we were interested in the effect of probe congruency. Given that the behavioral analysis did not show any differences in the strength of the congruency effect between the single‐feature conditions, the data were collapsed across the visual and the auditory conditions. For both the anterior and posterior clusters, the time‐frequency data were submitted to a cluster‐based permutation test contrasting congruent versus incongruent probe trials. The statistical analysis included all frequencies and all time points between the onset of the probe item and 1000 ms following the offset of the probe (3800–5000 ms). Earlier time points were not considered because “congruency” was not known prior to the appearance of the probe. The conjunction condition was excluded from this analysis because both features were task‐relevant. Finally, to test for an interaction between probe congruency and set size, analogous to the behavioral data analysis, the difference between congruent versus incongruent probe trials was computed separately for set sizes 1 and 2 trials and submitted to a cluster‐based permutation test, including the same frequencies and time points as outlined above.

#### 2.9.4 Cluster‐Based Permutation Tests

The fieldtrip‐implemented cluster‐based permutation test adheres to the following procedure: For each time‐frequency pair, a two‐sided paired‐sample *t*‐test was conducted, contrasting the experimental conditions of interest. Only sampling points that yielded a *p*‐value below 0.025 were considered for the subsequent clustering stage. At this stage, selected samples were clustered based on temporal and spectral adjacency. The sum of all *t*‐values served as the cluster‐level statistic for each identified cluster. If multiple clusters were identified, the maximum cluster‐level statistic was selected for further evaluation of the statistical significance. Significance probabilities were calculated by using the Monte Carlo method. That is, all trials are randomly assigned a condition label (e.g., visual vs. auditory vs. conjunction conditions to test for the main effect of the condition). Following the random partitioning, a two‐sided paired‐sample *t*‐test was conducted for each time‐frequency pair. This random assignment of condition labels and the subsequent calculation of test statistics was repeated 1000 times, resulting in time points × frequencies × participants × permutations matrix of cluster‐test statistics. The observed test statistic is then compared to a threshold obtained from this distribution of test statistics under the null hypothesis. Differences between conditions were considered significant if the observed test statistic was lower than 1st or larger than the 99th percentile of the distribution of significance probabilities resulting from the permutation procedure. Please note that descriptions of the time range or frequency range comprised in a given cluster should not be interpreted as definite time or frequency boundaries of the true effect, given that what is tested is the size of a cluster rather than exact time‐frequency point differences between conditions (Sassenhagen and Draschkow 2019).

## 3 Results

### 3.1 Behavioral Results

#### 3.1.1 Is the Task‐Irrelevant Feature of an Attended Object Stored in Working Memory?

RTs and accuracy differences between congruent and incongruent probe trials served as the dependent variables for two separate rmANOVA, including the factors condition (auditory, visual) and set size (set size 1 vs. 2). The degree of interference by task‐irrelevant incongruent probe features serves as an index of their storage in working memory.

**FIGURE 3:**
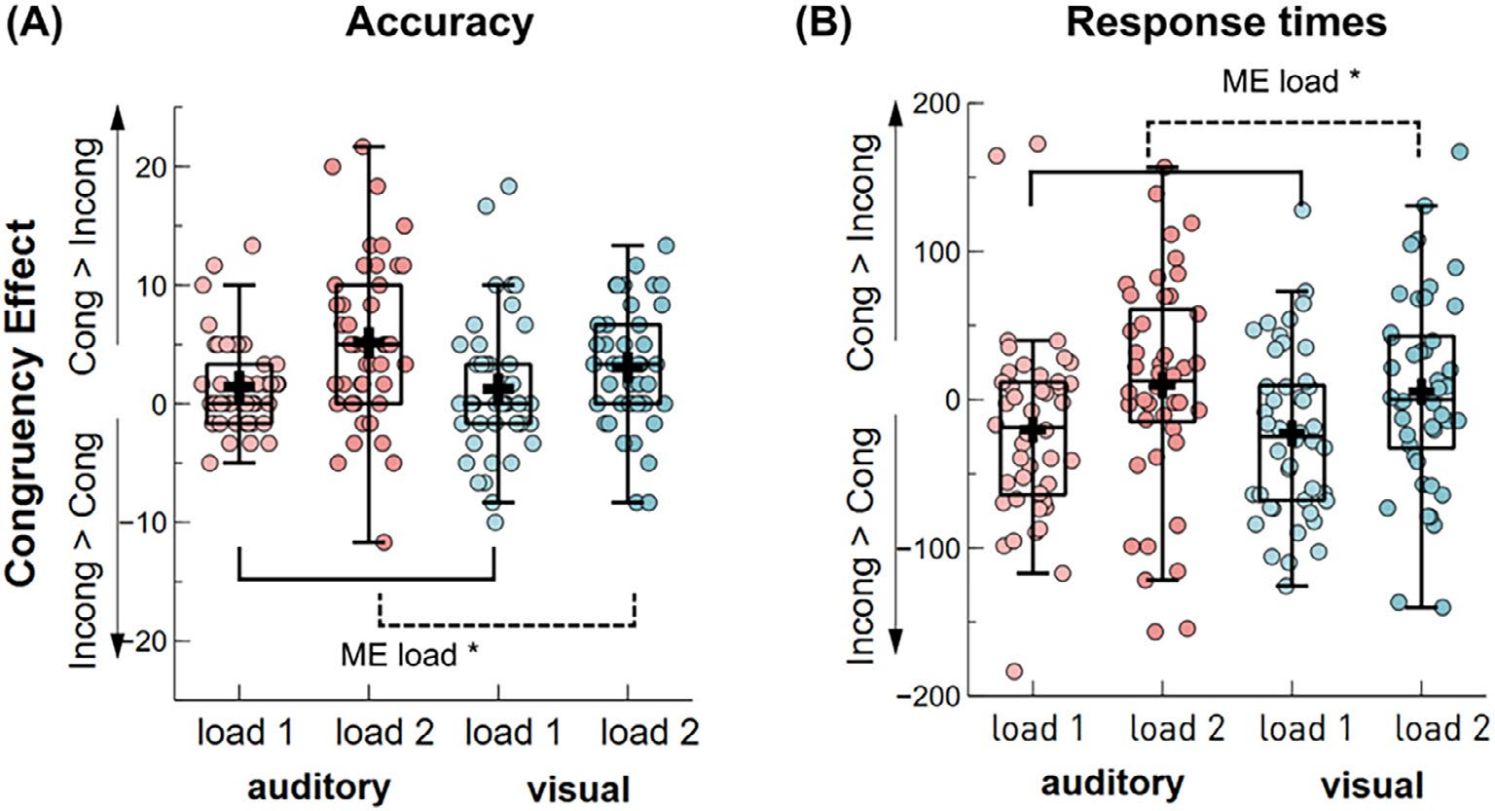
Probe congruency effect between single‐feature conditions for different set size trials. (A) Proportion of correct responses for the congruency effect (congruent—incongruent) between auditory and visual conditions; (B) RTs for the congruency effect between auditory and visual conditions. Boxplots show the ±1.5 interquartile range and the median. The dots illustrate individual participant averages per condition. A black cross illustrates the condition mean. *p < 0.05.

For accuracy, a significant main effect of set size, *F*(1, 43) = 13.39, *p* = 0.008, 𝜂^2^_*p*_ = 0.24, BF_10_ = 19.71, pointed out greater accuracy differences between congruent versus incongruent trial types in set size 2, compared to set size 1 trials (see Figure 3A). Additional paired‐samples *t*‐tests revealed that, compared to incongruent trials, participants scored higher in congruent probe trials for both set size 1, *t*(43) = 2.41, *p*_corr_ = 0.02, *d* = 0.36, BF_10_ = 2.16, and set size 2, *t*(43) = 6.11, *p*_corr_ = 0.002, *d* = 0.92, BF_10_ > 1000. There was neither a significant interaction of condition and set size, *F*(1, 43) = 1.47, *p* = 0.23, 𝜂^2^_*p*_ = 0.033, BF_10_ = 0.56, nor a main effect of condition, *F*(1, 43) = 1.91, *p* = 0.17, 𝜂^2^_*p*_ = 0.04, BF_10_ = 0.42.

For RTs, there was a main effect of set size, *F*(1, 43) = 7.83, *p* = 0.008, 𝜂^2^_*p*_ = 0.15, BF_10_ = 5.08, pointing out greater RTs differences between congruent versus incongruent trial types in set size 1 compared to set size 2 trials (see Figure 3B). Additional paired‐samples *t*‐tests revealed that, compared to incongruent trials, participants performed faster in congruent probe trials in set size 1, *t*(43) = −3.05, *p*_corr_ = 0.008, *d* = −0.46, BF_10_ = 8.96. This congruency effect vanished in set size 2 trials, *t*(43) = 0.93, *p*_corr_ = 0.36, *d* = 0.14, BF_10_ = 0.25. Neither the main effect of condition, *F*(1, 43) = 0.17, *p* = 0.69, 𝜂^2^_*p*_ = 0.004, BF_10_ = 0.21, nor the interaction of condition and set size, *F*(1, 43) = 0.009, *p* = 0.93, 𝜂^2^_p_ = 0.002, BF_10_ = 0.70, were significant.

A supplementary analysis revealed that the probe congruency effect in terms of accuracy is even stronger when participants are attending to both features (i.e., in the conjunction condition) compared to when attending to visual, *F*(1, 43) = 6.82, *p* = 0.012, 𝜂^2^_*p*_ = 0.06, BF_10_ = 3.72, or auditory features, *F*(1, 43) = 36.51, *p* < 0.001, 𝜂^2^_*p*_ = 0.28, BF_10_ > 1000 (For further details, the reader is referred to the Supporting Information, Section 3).

Overall, incongruent probes decreased accuracy in both set size conditions and increased RTs in one‐item trials, irrespective of whether participants were attending to visual or auditory features. This suggests that task‐irrelevant features are stored in working memory to some degree, regardless of the task goals. However, the data do not fully support the hypothesis that the degree of interference would decrease with increasing working memory load.

#### 3.1.2 Is the Set Size Effect Driven by the Number of Features or by the Number of Objects?

**FIGURE 4:**
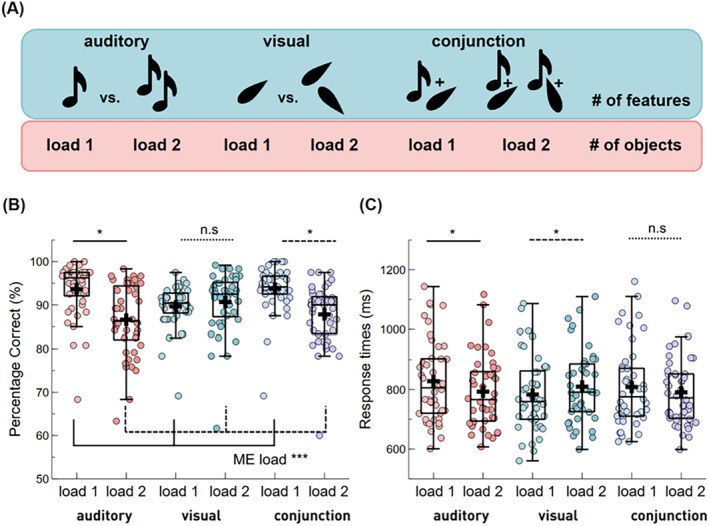
Set size effect across memory conditions. (A) The upper row shows how many task‐relevant features each set size (i.e., set size 1 vs set size 2) included per condition. The lower row illustrates how many objects each set size included per condition; (B) The proportion of correct responses varied by set size across memory conditions; (C) Reaction times varied by set size across memory conditions. Boxplots show the ±1.5 interquartile range and the median. The dots illustrate individual scores per condition. A black cross illustrates the condition mean. *p < 0.05, ***p < 0.001, n.s., not significant.

In the single‐feature conditions, the number of task‐relevant features was one or two in set size 1 and set size 2 trials, respectively. In contrast, the number of task‐relevant features in the conjunction condition was two or four in set size 1 and 2 trials, respectively (see Figure 4A). Thus, if only task‐relevant information is stored in a feature‐based fashion, the set size effect in the conjunction condition should be greater than in the single‐feature conditions. In contrast, the number of objects is always one versus two in all three memory conditions (auditory, visual, and conjunction). Hence, a comparable set size effect across conditions would favor object‐based storage.

**FIGURE 5:**
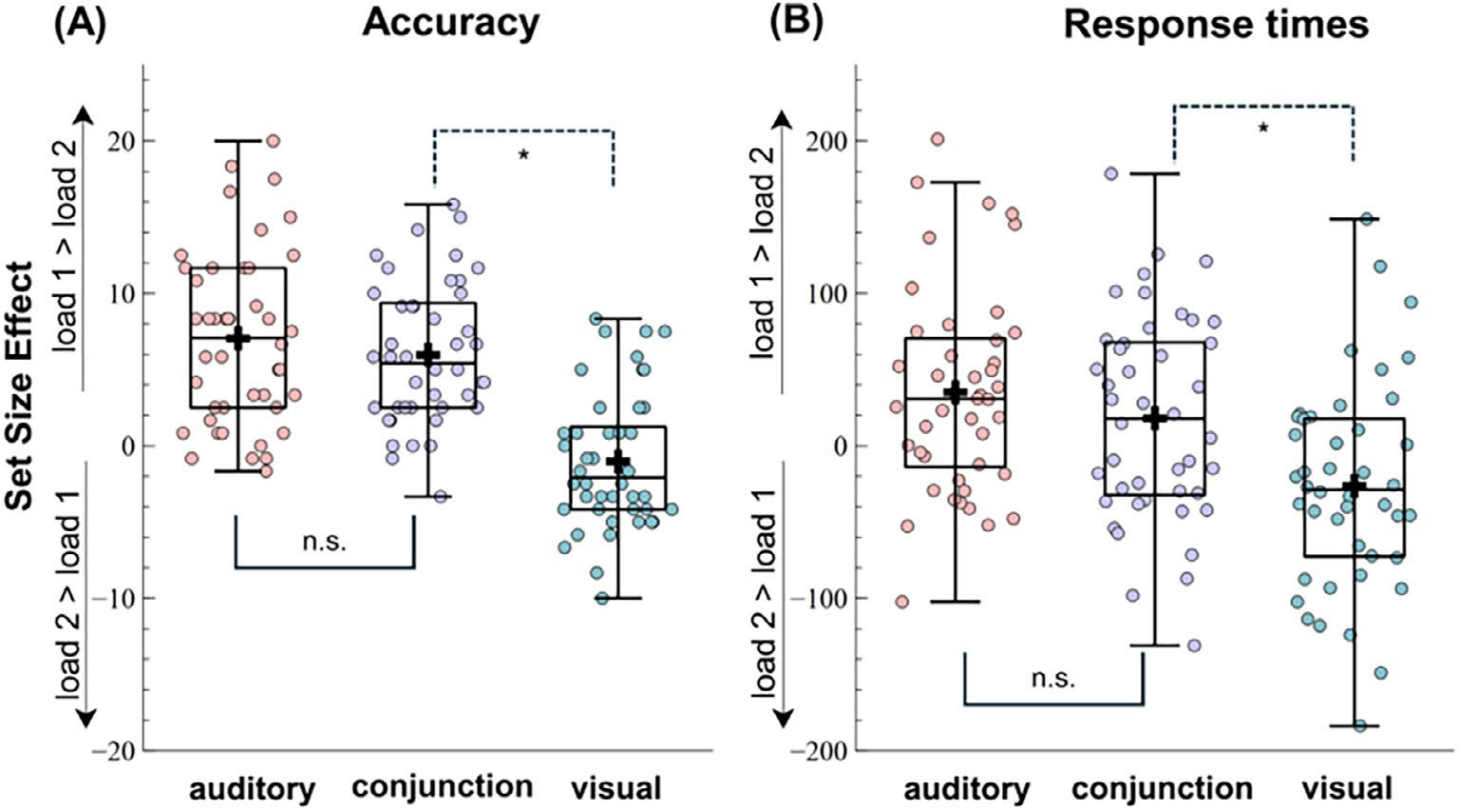
Set size effect between single‐feature and conjunction conditions. (A) Proportion of correct responses for the set size effect (load 1–load 2) between single feature and conjunction conditions; (B) RTs for the set size effect between single feature and conjunction conditions. Boxplots show the ±1.5 interquartile range and the median. The dots illustrate individual participant averages per condition. A black cross illustrates the condition mean. *p < 0.05, n.s., not significant.

A 3 (condition) × 2 (set size) rmANOVA revealed a main effect of set size, with greater accuracy for set size 1 compared to set size 2 trials, *F*(1, 43) = 58.74, *p* = < 0.001, 𝜂^2^_*p*_ = 0.58, BF_10_ > 1000, and a significant interaction between set size and condition, *F*(2,1.35) = 43.58, *p*_corr_ = < 0.001, 𝜂^2^_*p*_ = 0.50, BF_10_ > 1000. Yet, accuracies did not differ between conditions, *F*(2,1.32) = 0.61, *p*_corr_ = 0.48, 𝜂^2^_*p*_ = 0.01, BF_10_ = 0.09 (see Figure 4B). To resolve the interaction, we contrasted the magnitude of the set size effect between conditions. Contrasting the set size effect in accuracy between the auditory and the conjunction condition did not reveal any significant differences, *t*(43) = 1.08, *p*_corr_ = 0.29, *d* = 0.16, BF_10_ = 0.28 (see Figure 5A). Contrasting the visual and the conjunction condition, however, the set size effect in accuracy was greater in the conjunction (*M* = 5.97, SD = 4.65) compared to the visual condition (*M* = −1.02, SD = 4.51), *t*(43) = −12.70, *p*_corr_ = 0.002, *d* = −1.92, BF_10_ > 1000 (see Figure 5A).

For RTs, a rmANOVA revealed no significant main effect of condition, *F*(2,1.44) = 1.69, *p*_corr_ = 0.20, 𝜂^2^_*p*_ = 0.04, BF_10_ = 0.25, or set size, *F*(1, 43) = 1.26, *p* = 0.27, 𝜂^2^_*p*_ = 0.03, BF_10_ = 0.34 (see Figure 4C). Similar to the accuracy results, there was a significant interaction of condition and set size for RTs, *F*(2,1.59) = 16.21, *p*_corr_ = < 0.001, 𝜂^2^_*p*_ = 0.27, BF_10_ = 23.97. Contrasting the set size effect between the auditory and the conjunction condition did not reveal any significant differences, *t*(43) = 1.46, *p*_corr_ = 0.15, *d* = 0.22, BF_10_ = 0.44 (see Figure 5B). However, the set size effect in RTs was greater in the visual (*M* = −26.42, SD = 68.31) compared to the conjunction condition (*M* = 18.06, SD = 67.72), *t*(43) = −5.53, *p*_corr_ = 0.002, *d* = −0.83, BF_10_ > 1000 (See Figure 5B).

Additional follow‐up analyses to verify the existence of a significant set size effect within each condition (see Table 1) showed that the opposing patterns for the visual versus conjunction comparisons in RT (visual > conjunction) and accuracy (visual < conjunction) are driven by the fact that in the visual condition the set size effect was only evident in response times, but absent in accuracy, while for the conjunction condition it was the other way around (i.e., significant set size effect in accuracy, but not in RTs).

**TABLE 1:** *t*‐Test results comparing set size 1 versus 2 for each memory condition.

Variable	Auditory							Visual							Conjunction						
	Set size 1		Set size 2		*t* (43)	*p*	Cohen's *d*	Set size 1		Set size 2		*t* (43)	*p*	Cohen's *d*	Set size 1		Set size 2		*t* (43)	*p*	Cohen's *d*
	*M*	SD	*M*	SD				*M*	SD	*M*	SD				*M*	SD	*M*	SD			
Accuracy (%)	93.6	6.18	86.6	8.26	8.18	0.003	1.23	89.6	4.93	90.7	6.84	1.50	0.14	0.23	93.8	5.31	87.8	6.91	8.52	0.002	1.28
RTs (ms)	828	136.6	793	120.7	3.47	0.003	0.52	783	126.2	809	120.3	−2.57	0.028	0.39	808	137.4	790	114.3	1.77	0.084	0.27

*Note:* Paired sample's *t*‐test results for the contrast between set size 1 versus 2 for each memory condition. *p*‐values are adjusted for multiple comparisons.

Abbreviations: *M*, mean; RTs, reaction times; SD, standard deviation.

In sum, with respect to the question of whether audiovisual object storage is feature‐ or object‐based, the data shows no perfectly consistent pattern. While auditory vs. conjunction contrast is in line with an object‐based account of working memory, the visual vs. conjunction contrast, at least in terms of accuracy, rather favors a feature‐based account.

### 3.2 ERP Data

**FIGURE 6:**
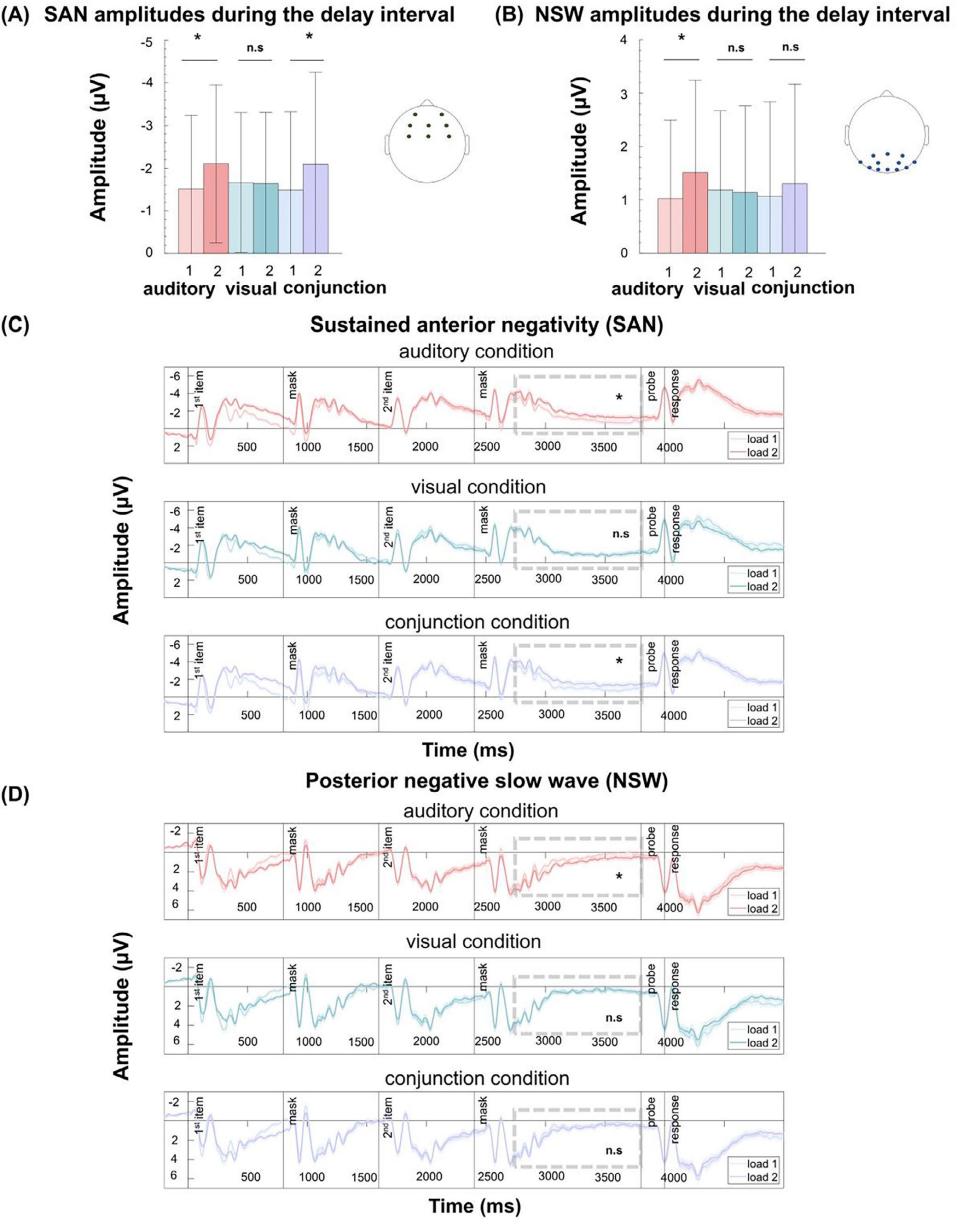
Condition‐specific, grand‐average ERP waveforms at anterior (SAN) and posterior (NSW) electrode sites. Mean ERP amplitudes in (A) and (B) show the SAN and NSW amplitudes during the maintenance interval for each memory condition for set sizes 1 and 2 trials (error bars show standard deviations). The topographies show the electrode clusters used for SAN (left panel) and NSW (right panel). The ERPs in (C) and (D) depict the time course of the SAN and NSW, respectively, separately for the auditory, visual, and conjunction conditions and for set sizes 1 and 2 trials. The gray rectangle indicates the corresponding analysis time window, spanning the maintenance period. Significant paired‐sample *t*‐test contrasts (i.e., auditory set size 1 vs. set size 2 and conjunction set size 1 versus set size 2 comparisons for SAN and auditory set size 1 versus set size 2 for NSW) were marked with an asterisk (*p* < 0.05). n.s., not significant.

ERP correlates of working memory storage were investigated to track the maintenance of task‐relevant and task‐irrelevant features (see Figure 6A,B for the respective electrodes used in the analyses).

A 3 (condition) × 2 (set size) rmANOVA of mean SAN amplitudes revealed a main effect of set size, *F*(1, 43) = 7.37, *p* = 0.010, 𝜂^2^_*p*_ = 0.15, BF_10_ = 2.91. There was neither a significant main effect of condition, *F*(1, 43) = 0.71, *p* = 0.49, 𝜂^2^_*p*_ = 0.02, BF_10_ = 0.08, nor a significant interaction, *F*(1, 43) = 2.54, *p* = 0.09, 𝜂^2^_*p*_ = 0.06, BF_10_ = 1.16. Despite the lack of a significant interaction, pre‐registered one‐sided paired‐samples *t*‐tests were conducted to verify the presence of a set size effect within conditions. Accordingly, set size 2 trials displayed significantly more negative SAN amplitudes than set size 1 trials in both the auditory, *t(*43) = 2.82, *p* = 0.007, *d* = 0.43, BF_10_ = 5.21, and the conjunction condition, *t*(43) = 2.26, *p* = 0.03, *d* = 0.34, BF_10_ = 1.60, in which auditory features were task‐relevant (see Figure 6A,C). However, no effect of set size was evident in the visual condition, *t*(43) = −0.12, *p* = 0.91, *d* = −0.02, BF_10_ = 0.16, where auditory features were task‐irrelevant. The corresponding Bayes factor provides moderate evidence for the null hypothesis, indicating that task‐irrelevant features are not actively maintained.

An analysis of mean NSW amplitudes revealed no significant effects (all *p* > 0.10). Again, despite the lack of a significant interaction, planned one‐sided paired‐sample *t*‐tests were conducted as pre‐registered. Contrary to our expectations, no significant set size variation of NSW amplitudes was evident in the visual, *t*(43) = 0.25, *p* = 0.81, *d* = 0.04, BF_10_ = 0.17, or the conjunction, *t*(43) = −0.98, *p* = 0.33, *d* = −0.15, BF_10_ = 0.26, condition, where visual features were task‐relevant (see Figure 6B,D). In the auditory condition, NSW amplitudes were significantly more positive in set size 2 trials than in set size 1 trials, *t*(43) = −2.65, *p* = 0.01, *d* = −0.4, BF_10_ = 3.53, exhibiting a reversed set size effect (see Figure 6D).

### 3.3 Time‐Frequency Results

#### 3.3.1 The Effect of Memory Condition

**FIGURE 7:**
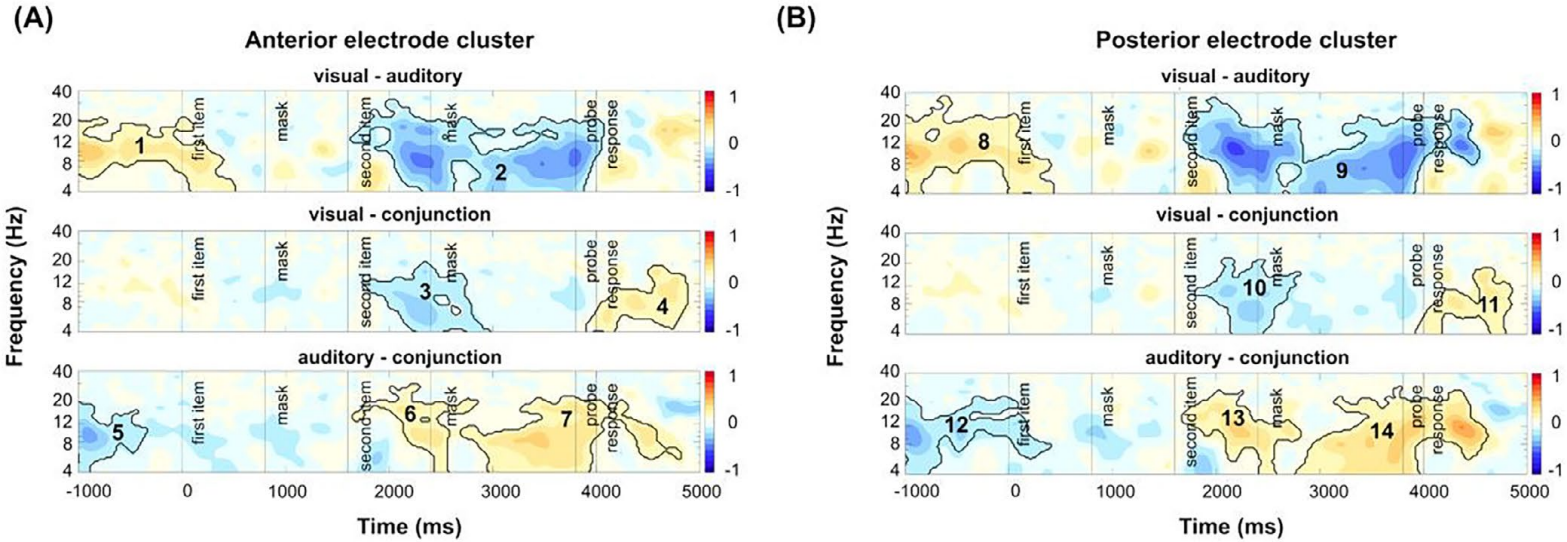
Cluster‐based permutation test results between memory conditions. Differences in oscillatory power between conditions were tested with cluster‐based permutation tests across (A) the midcentral electrode cluster and (B) the parieto‐occipital electrode cluster. Solid lines illustrate significant clusters with a *t*‐mass value smaller than 1st or larger than the 99th percentile of the distribution of significance probabilities of the null distribution.

Cluster‐based permutation analyses, contrasting the pair‐wise combinations of memory conditions, revealed significant differences between conditions in the encoding period after the second‐item presentation, in the maintenance interval, as well as during probe presentations. Figure 7 shows the time course of the condition differences and the obtained clusters. Critically, the pattern of results was highly comparable for the anterior and the posterior clusters (see Figure 7A,B). For an overview of the time course of oscillatory activity within the individual conditions, please see Figure S4 in the Supporting Information.

In the encoding period following the second‐item presentation, differences between the conditions appear most prominent in the alpha band but expand into the adjacent theta and beta bands. Specifically, the contrast between the visual and the conjunction condition shows a relative increase in power in the conjunction condition following the second memory item. The corresponding cluster (cluster 3 in anterior electrodes: *p* = 0.001, *d* = −0.22 and cluster 10 in posterior electrodes: *p* = 0.001, *d* = −0.19) comprises the theta‐ and alpha range while also extending into the lower beta‐band (anterior electrodes: 4 to 19 Hz, posterior electrodes: 4–22 Hz). In the time domain, the cluster in the observed data extends from 1740 to 2970 ms (relative to first item‐onset) at anterior electrodes and from 1800 to 2800 ms at posterior electrodes. In the response period, the cluster‐based permutation analysis also revealed a relatively stronger power suppression in the conjunction condition compared to the visual condition. The corresponding cluster (cluster 4 in anterior electrodes: *p* = 0.001, *d* = 0.17, cluster 11 in posterior electrodes: *p* = 0.001, *d* = 0.16) mainly comprises the theta‐ and alpha‐band (anterior electrodes: 4–18 Hz, posterior electrodes: 4–18 Hz) and extends roughly from 3910 to 4870 ms at anterior and from 3930 to 4850 ms at posterior electrodes.

When comparing the auditory and the conjunction conditions, a relative increase in power can be observed in the auditory compared to the conjunction condition in the encoding period following the second‐item presentation. The corresponding cluster in the observed data (cluster 6 in anterior electrodes: *p* = 0.001, *d* = 0.32, cluster 13 in posterior electrodes: *p* = 0.003, *d* = 0.28) comprises the alpha‐band but extends into the theta and lower beta‐band (anterior electrodes: 4–29 Hz, posterior electrodes: 4–27 Hz). A second cluster (cluster 7 in anterior electrodes: *p* = 0.001, *d* = 0.45, cluster 14 in posterior electrodes: *p* = 0.001, *d* = 0.47) emerges in the maintenance period and extends into the recall period (anterior electrodes: 2700–4800 ms, posterior electrodes 2760–4610 ms). Again, the cluster spans a broad range of frequencies in the theta, alpha, and lower beta‐band (anterior electrodes: 4–22 Hz, posterior electrodes: 4–29 Hz). The latter cluster indicates a relative increase in power in the auditory compared to the conjunction condition, or in other words, a relatively stronger decrease in power in the conjunction condition.

Finally, when contrasting the auditory and the visual conditions, the cluster test reveals a broad cluster (cluster 2 in anterior electrodes: *p* = 0.001, *d* = −0.57; cluster 9 in posterior electrodes: *p* = 0.001, *d* = −0.56) that spans the encoding interval after second‐item presentation, the maintenance period, and the response interval (anterior electrodes: 1630–4060 ms, posterior electrodes 1700–4530 ms). In the frequency dimension, the effect appears most prominent in the alpha‐band but clearly extends broadly in the adjacent theta and beta band (anterior electrodes: 4–32 Hz, posterior electrodes: 4–35 Hz). This cluster reflects a sustained relative increase in power in the auditory condition compared to the visual condition.

Lastly, the cluster tests also revealed significant differences in the baseline period, which we mainly attributed to the block‐wise presentation of conditions. Namely, there was a stronger anticipatory suppression of alpha power in the auditory condition compared to the conjunction and visual conditions, respectively. In the former case, the corresponding cluster (cluster 5 in anterior electrodes: *p* = 0.001, *d* = −0.72, cluster 12 in posterior electrodes: *p* = 0.001, *d* = −0.71) comprised time points from −1280 to −350 ms at anterior electrodes and from −1280 to 400 ms at posterior electrodes. In the frequency domain, the cluster spanned predominantly the alpha band but expanded into the adjacent theta and lower beta‐band (anterior electrodes: 4–30 Hz, posterior electrodes: 4–28 Hz). In the latter case, the corresponding cluster (cluster 1 in anterior electrodes, *p* = 0.001, *d* = 0.69, cluster 8 in posterior electrodes: *p* = 0.001, *d* = 0.72) comprised time points from −1280 to 505 ms at anterior electrodes and from −1280 to 420 ms at posterior electrodes. In the frequency domain, the effect was most prominent in the alpha band, extending through theta and beta bands (anterior electrodes: 4–22 Hz, posterior electrodes: 4–36 Hz). Notably, the clusters in the poststimulus period cannot be due to those baseline differences, as the direction of effects in the baseline is reversed compared to those found later in the trial.

In summary, the three pair‐wise contrasts outlined above show a prominent relative increase in alpha power (and adjacent frequency bands) in auditory blocks compared to visual and conjunction blocks in the encoding and maintenance period. Further, a relative increase in alpha power (as well as adjacent frequency bands) was evident in the conjunction compared to visual blocks during maintenance. Finally, a consistent decrease in alpha power (and adjacent frequency bands) in the conjunction condition was observed at recall compared to both single‐feature conditions.

#### 3.3.2 The Effect of Memory Load

**FIGURE 8:**
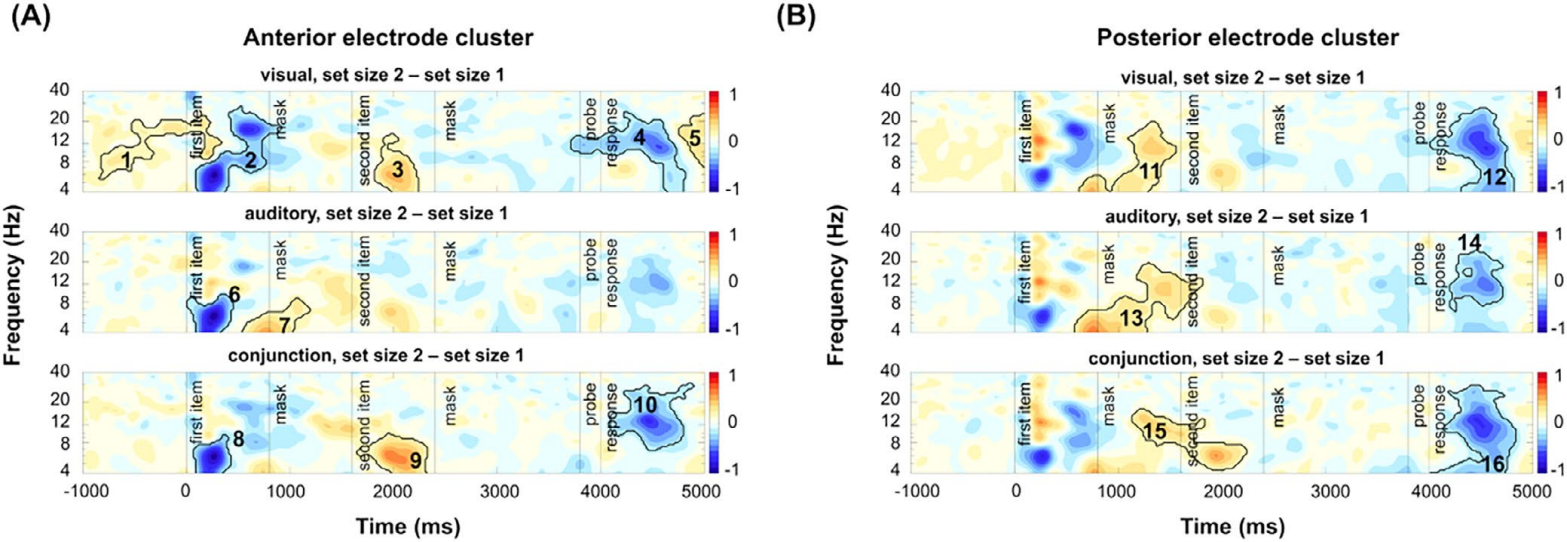
Cluster‐based permutation test results for set size 2 versus 1 contrast across memory conditions. Pair‐wise contrasts (between set size 2 vs. set size 1 trials) for time‐frequency power between conditions were tested with cluster‐based permutation tests for (A) the mid‐central electrodes and for (B) the parieto‐occipital electrodes. Solid lines illustrate significant clusters with a *t*‐mass value smaller than 1st or larger than the 99th percentile of the distribution of significance probabilities of the null distribution.

Figure 8 shows the pair‐wise combinations of set size 2 versus 1 trial within each memory condition for the anterior (see Figure 8A) and the posterior electrode cluster (see Figure 8B). Cluster‐based permutation analyses showed three major sets of functionally compatible clusters: (i) In the first few hundred milliseconds of the trial, set size 2 trials elicit a stronger decrease in theta power compared to set size 1 trials. This effect is only significant at the anterior electrode cluster (visual: *p* = 0.001, *d* = −0.75, auditory: *p* = 0.002, *d* = −0.53, conjunction: *p* = 0.007, *d* = −0.55) and extends to later time points (~900 ms) and higher frequencies (~4–28 Hz) in the visual condition. This set of clusters (see clusters 2, 6, and 8) can be attributed to differences in processing between the filler item in set size 1 trials and the encoding of a memory item in set size 2 trials and is thus not of major interest here.

(ii) Following the presentation of the first mask stimulus, a relatively sustained (~700–1600 ms) broad‐band power increase spanning the theta, alpha, and lower beta‐band is evident in set size 2 compared to set size 1 trials. This effect (see clusters 11, 13, and 15) is predominantly reflected at posterior electrode sites in the visual (*p* = 0.001, *d* = 0.40), auditory (*p* = 0.001, *d* = 0.50), and conjunction condition (*p* = 0.002, *d* = 0.37; but see also cluster 7 for a slightly earlier, but likely related cluster in the auditory condition at anterior electrode sites). This set of effects is followed by a relative increase in theta power in set size 2 compared to set size 1 trials. Correspondingly, significant clusters are in the visual condition (*p* = 0.001, *d* = 0.35) and in the conjunction condition (*p* = 0.001, *d* = 0.44) at anterior electrode sites (clusters 3 and 9, respectively). These two sets of clusters can—again—be attributed to differences in required cognitive control and attentional processing when processing the filler item in set size 1 trials compared to the storage and maintenance of the first memory item in set size 2 trials. Again, these effects were not the main objective of this investigation.

(iii) Finally, at recall, set size 2 trials consistently result in a stronger decrease in alpha power (and adjacent frequencies) compared to set size 1 trials. Corresponding, significant clusters were identified in all memory conditions and at both electrode sites (anterior electrode sites: visual condition: *p* = 0.001, *d* = −0.31, conjunction condition: *p* = 0.001, *d* = −0.31; posterior electrode sites: visual condition: *p* = 0.001, *d* = −0.32, auditory condition: *p* = 0.002, *d* = −0.24, conjunction condition: *p* = 0.001, *d* = −0.32), except for the auditory condition at the anterior electrode site (*p* = 0.013). This set of clusters (see clusters 4, 10, 12, 14, and 16) can be attributed to greater attentional demands when retrieving two‐item working memory representations compared to one‐item working memory representation.

Unlike other contrasts, a significant cluster in the baseline period of the visual condition was observed at anterior electrodes (cluster 1). However, it was not possible to prepare for a two‐item trial due to the randomization between different set‐size trials. Thus, this effect was not further interpreted.

In summary, differences at early stages point to the processing of the filler item in one‐item trials compared to the encoding of the first memory item in two‐item trials. Critically, the relative power increase in theta range might point out filler‐item processing, which is later discarded, as addressed by a relative decrease in theta range. Furthermore, stronger alpha power suppression at recall addresses greater attentional demands when retrieving two‐item working memory representations compared to one‐item working memory representations.

#### 3.3.3 The Effect of Probe Congruency

**FIGURE 9:**
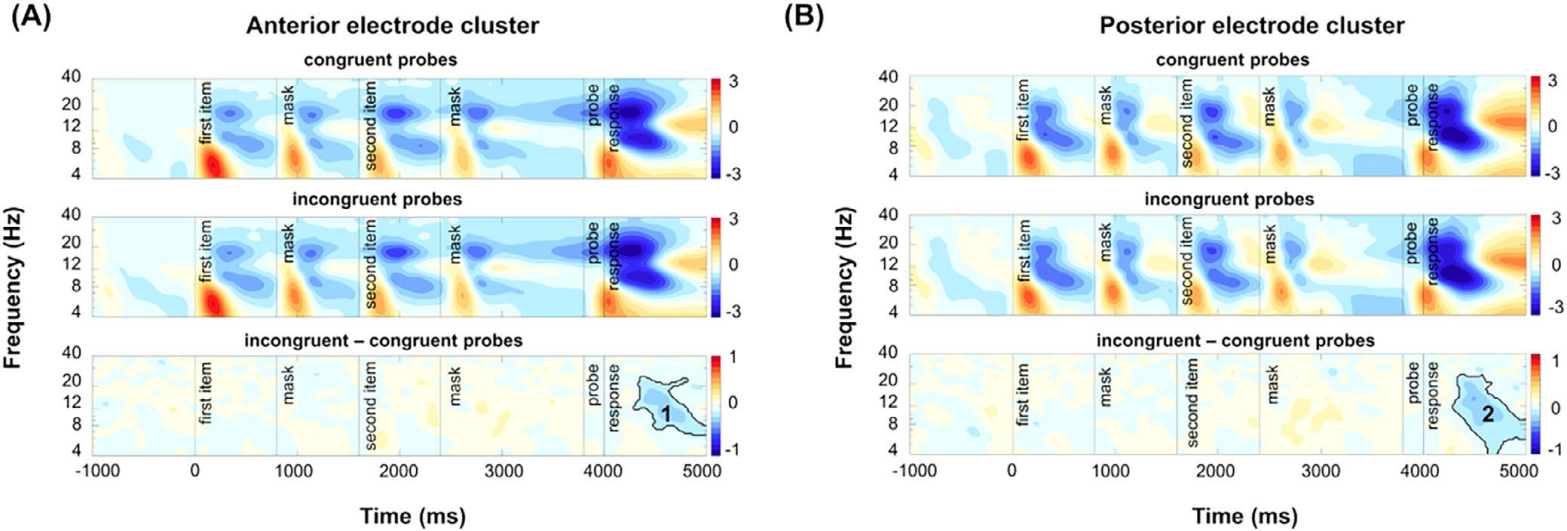
Time‐frequency modulations and cluster‐based permutation test results for incongruent versus congruent probe contrast. (A) Power was averaged across the midcentral electrode cluster for congruent and incongruent trials (upper and middle rows). Differences between the two conditions were tested with cluster‐based permutation tests (lower row); (B) Power was averaged across the parieto‐occipital electrodes for congruent and incongruent trials (upper and middle rows). Differences between the two conditions were tested with cluster‐based permutation tests (lower row). Solid lines illustrate significant clusters with a *t*‐mass value smaller than 1st or larger than the 99th percentile of the distribution of significance probabilities of the null distribution.

Furthermore, we were interested in the effect of probe congruency. This was an exploratory analysis as a followup on the interaction between these factors in behavioral performance. Since the behavioral analysis did not show an interaction of probe congruency and condition, the data were averaged across the two single‐feature conditions. Figure 9 shows a probe congruency effect on time‐frequency modulations, indicated by a stronger alpha power suppression following incongruent probes, compared to congruent probes. The corresponding cluster in the observed data (cluster 1 in anterior electrodes: *p* = 0.001, *d* = 0.08, cluster 2 in posterior electrodes: *p* = 0.001, *d* = −0.1) comprises the alpha‐band but extends into the theta and lower beta‐band (anterior electrodes: 7–24 Hz, posterior electrodes: 4–24 Hz), sustained between 4290 and 5000 ms at anterior and 4290 and 5000 ms at posterior electrodes. This result suggests a higher need for attentional control for incongruent probes, also supported by behavioral performance decay. The analysis did not show any significant interaction between set size and probe congruency (cluster in anterior electrodes: *p* = 0.31, cluster in posterior electrodes: *p* = 0.22).

## 4 Discussion

Previous research investigating the concurrent storage of auditory and visual features in working memory has predominantly adopted a dual‐task approach, considering the two modalities as competitors for a shared underlying resource. Here, we introduce a novel multisensory delay‐match‐to‐sample paradigm, in which bottom‐up integration of audiovisual features is promoted by spatiotemporal synchrony. The paradigm included three variants of task‐instructions: a cross‐modal conjunction condition, in which participants needed to maintain audiovisual objects, as well as two single‐feature conditions, in which participants attended to visual or auditory features, respectively. The present study reveals three major findings: First, we show that when attending to only one modality dimension of a compound audiovisual object, task‐irrelevant features are encoded into working memory, as indexed by interference with behavioral performance. Although ERP results revealed that the task‐irrelevant features of attended objects are not actively maintained in working memory, they tax attentional resources, as shown by alpha power dynamics during recall. Secondly, our behavioral results corroborate the notion that audiovisual working memory storage is both feature‐ and object‐based. Finally, we provide novel insights into the oscillatory dynamics underlying audiovisual working memory. We show that attentional resources, supporting linking between auditory and visual modalities, are specifically recruited at recall rather than during maintenance or encoding. In the following, we discuss how the current findings advance our understanding of multisensory interactions in working memory.

### 4.1 Are Task‐Irrelevant Cross‐Modal Features Automatically Encoded and Stored in Working Memory?

In the present study, the manipulation of probe congruency allowed us to investigate whether task‐irrelevant features of an audiovisual object are encoded into working memory, when participants attend to only one of the two concurrently present modalities. Previous work had shown that when a unimodal object is attended, attention spreads toward a task‐irrelevant but simultaneously occurring object from a different modality (Busse et al. 2005; Donohue et al. 2011). Critically, here we show for the first time that the effect of this cross‐modal spread of attention during encoding persists throughout the maintenance interval of an audiovisual working memory task. This was indexed by the interference of task‐irrelevant cross‐modal features at recall, suggesting that the task‐irrelevant features were still represented to some degree. This finding aligns with previous work in the visual working memory domain, showing that task‐irrelevant features are not entirely dropped from working memory (Schneider et al. 2015, 2016), but can still be retrieved, albeit only with low precision (Shin and Ma 2016), as well as with evidence, demonstrating that task‐irrelevant visual features can be decoded based on the multivariate EEG signal (Chen et al. 2021; but see also Yu and Shim 2017, Bocincova et al. 2017). Notably, a very similar behavioral interference effect was reported in an auditory working memory study by Joseph et al. (2015), in which participants maintained sequences of whole auditory objects or were instructed to memorize only one of their component features (i.e., a spectral feature or a temporal feature). The authors found that performance was reduced when the task‐irrelevant dimension of the memory objects and the probe varied randomly compared to when it was held constant. They attribute this effect to the interference of the irrelevant with the relevant dimension as well as a feature extraction cost. Notably, the authors emphasize that interference could occur both at encoding, where interference of the irrelevant dimension may add noise to the memory representation of the task‐relevant feature, as well as at recall, where participants need to focus on the probe's relevant dimension while ignoring the task‐irrelevant dimension. While we cannot fully exclude that the congruency effect in the present study is partially due to interference already at encoding, the observed alpha power dynamics at recall for congruent as compared to incongruent probes provide evidence for interference at recall. Specifically, a stronger suppression of alpha power was evident during recall when participants responded to an incongruent compared to a congruent probe, reflecting greater attentional demands when handling incongruency. Here, for a congruent probe (i.e., both the task‐relevant and the task‐irrelevant probe features suggest the same response), a working memory representation containing both visual and auditory features can serve as a template to successfully reject or accept the probe object as a whole without causing any interference. In contrast, when confronted with an incongruent probe, where processing the task‐irrelevant modality would result in the selection of a false response (e.g., the task‐relevant feature dimension suggests a “yes” response whereas the task‐irrelevant feature dimension suggests a “no” response), it requires additional attentional resources to selectively compare only the task‐relevant features of the probe to the contents of working memory.

Notably, despite behavioral evidence for the persistence of a memory trace reflecting the task‐irrelevant cross‐modal feature, an ERP analysis did not provide any evidence for the active maintenance of auditory features in the attend‐visual condition. Critically, Bayesian statistics provided evidence for the lack of a load‐modulation of auditory SAN amplitudes in the visual condition. In contrast, in the auditory and the conjunction condition, where auditory features were task‐relevant, SAN amplitudes increased with memory load, in line with previous studies, establishing the SAN as an indicator of auditory working memory storage (Alunni‐Menichini et al. 2014; Guimond et al. 2011). The apparent contradiction between the behavioral and ERP results can be reconciled by models which posit that working memory content can be maintained by neural activity in an “activity‐silent mode” (Stokes 2015). However, it should also be noted that we were not able to find the hypothesized memory load modulations of posterior NSW amplitudes as an index of visual working memory storage, irrespective of whether visual information was task‐relevant or irrelevant. This could be due to some prominent differences in our study design compared to previous studies, exploiting NSW ERP effects. The majority of previous studies (Diaz et al. 2021; Feldmann‐Wüstefeld 2021; Fukuda et al. 2015) used concurrent stimulus presentation, as compared to sequential presentation in the present study, and higher set sizes (up to set size 8). Sequential presentation of visual items was shown to be less susceptible to forgetting compared to concurrent stimulus presentation (Ricker and Cowan 2014), which might explain the lack of a behavioral set size effect in the visual condition, contributing to the lack of an NSW amplitude modulation. Finally, it remains possible that integrated audiovisual working memory items undergo some transformation which is not fully captured by ERP correlates for unimodal working memory storage. On a different note, the observed discrepancy between the visual and auditory condition in terms of behavioral and ERP‐based set size effects further resonates with previous work, suggesting that one item of auditory information may place a greater load on working memory than one item of visual information (Fougnie and Marois 2011; Saults and Cowan 2007).

### 4.2 Are Audiovisual Features Stored as an Integrated Multisensory Object?

Overall, the evidence discussed so far is in line with the assumption that audiovisual features are automatically integrated in a bottom‐up fashion under conditions of audiovisual synchrony and low perceptual load (reviewed by Talsma et al. 2010) and subsequently maintained in a potentially activity‐silent format (Stokes 2015) or outside the focus of attention (Oberauer 2002; Oberauer and Lin 2017). Although, on a cautionary note, it should be mentioned that the present study did now allow for an assessment of gamma power (reviewed by Engel et al. 2012) or ERP modulations according to an additive model (Besle et al. 2004) as explicit evidence for multisensory integration during encoding. Thus, despite clear evidence that both auditory and visual features are stored in working memory, in at least a residual form, in the two single‐feature conditions, a critical question that remains to be addressed is whether they are stored in an integrated format or rather in separate stores. We postulate that in the present paradigm, an audiovisual representation is formed at encoding, irrespective of the task instructions. That is because we explicitly designed the task to facilitate bottom‐up encoding. In accordance with the notion of a hierarchical working memory structure (Brady et al. 2011), we assume that this representation contains both a feature‐ and an object level, allowing participants to focus limited working memory resources on the maintenance of the feature‐level in the single‐feature conditions while relying more strongly on the (integrated) object level in the conjunction condition. Corroborating the assumption of a hierarchical working memory representation on the audiovisual level (Li et al. 2022), the behavioral analysis of set size effects showed evidence for both object‐based as well as feature‐based storage. Critically, in line with the above model, we show that the congruency effect (i.e., reduced accuracy in incongruent compared to congruent trials) in the conjunction condition is considerably larger compared to the single‐feature conditions (see Supporting Information, Section 3). This suggests that focusing on audiovisual objects in the conjunction condition might come with a cost of “breaking up” this integrated template in case of an incongruent probe type. On the contrary, disentangling the congruency effect within conditions shows that correct responses to incongruent probes in the conjunction condition were faster compared to responses to congruent probes. Thus, in correct trials, switching to the feature‐dimension of the representation appeared to not come at a cost. On the neural level, we do not find conclusive evidence for the maintenance of an integrated working memory representation. The oscillatory dynamics (see also “The effect of memory condition”) during the maintenance period rather suggest domain‐specific modulations, while integration across modalities appears to occur specifically during recall. This could be more broadly in line with the distinction between cross‐modal *binding* as a purely stimulus‐driven, perceptual process, and multisensory *integration* at the level of decision making, made by Bizley et al. (2016). Ultimately, a conclusive answer to the question of whether audiovisual working memory storage relies on an integrated representation warrants further investigation.

### 4.3 Oscillatory Dynamics Underlying Audio‐Visual Working Memory

An additional central finding concerns the underlying oscillatory signatures of multisensory working memory. While previous studies have primarily focused on the neurocognitive mechanisms supporting the prioritization of one modality over the other (e.g., Spitzer and Blankenburg 2012; van Ede et al. 2017), the present study allowed us to track oscillatory mechanisms involved in audiovisual working memory. Here, by contrasting the single‐feature conditions with the conjunction condition, we adopted the following logic: Any mechanism reflecting the linking of information across modalities should be consistently evident for both comparisons of interests (auditory vs. conjunction; visual vs. conjunction). This was only the case in the recall phase, where we coherently observed stronger alpha‐beta power suppression over the frontal and parieto‐occipital cortex in the conjunction condition compared to both single‐feature conditions. That is, when the task requires an audiovisual judgment, additional attentional resources seem to be recruited via the fronto‐parietal attentional network specifically at recall rather than during maintenance or encoding. This extends previous reports of stronger alpha‐ and beta‐power suppression accompanying recognition judgments in a cross‐modal delayed‐match‐to‐sample paradigm, requiring participants to compare a sample angle encoded in one modality (i.e., a sample angle either encoded in visual or kinesthetic modality) to a probe angle in either the same or a different modality (Seemüller et al. 2012). On a behavioral level, however, we do not find an analogous effect of the stronger alpha/beta power suppression in the conjunction condition, addressing additional attentional demands compared to single‐feature conditions.

Further, in the late encoding period and during maintenance, we observed differential modulations of oscillatory power, most prominent in the alpha‐band but extending into the adjacent theta and lower beta‐band. Essentially, as the prioritization shifts from a focus on purely visual features to purely auditory features, with the conjunction condition residing in‐between, we observe a stronger relative increase in alpha power over posterior scalp sites. In line with the postulated inhibitory function of alpha oscillations (Jensen and Mazaheri 2010; Klimesch et al. 2011), this pattern of results aligns well with the notion of a progressive disengagement of visual areas as participants have to re‐distribute attentional resources between modalities (i.e., conjunction condition) or focus exclusively on auditory features (i.e., auditory condition). Notably, we also observed a very similar pattern of results at a fronto‐central cluster of electrodes. Although these electrodes were selected based on a task‐independent localizer procedure, aiming at identifying electrodes that were maximally sensitive to auditory stimuli, due to methodological constraints and anatomical characteristics of the auditory cortex (Clements et al. 2023), it is unlikely that the observed power modulations reflect auditory oscillations. However, consistent with our results, modulations of fronto‐central alpha power have been previously observed in a cross‐modal matching paradigm. Specifically, Misselhorn et al. (2019) observed a relative increase in power for audiovisual compared to visual‐tactile attention. The authors postulate that frontal alpha oscillations reflect the origin of top‐down control.

More generally, to appreciate the novelty of the present findings, it is important to again highlight that despite the ample number of studies investigating neural oscillations in the context of multisensory processing and attention on a perceptual level (for a review, see Keil and Senkowski 2018), to the authors' best knowledge, this is the first study that explicitly exploits oscillatory mechanisms in an audiovisual working memory paradigm, with a specific focus on interactions of multisensory integration and multisensory (rather than inter‐sensory) attention in working memory.

## 5 Conclusion

Exploiting both behavioral as well as EEG data, the present study provides novel insights into interactions of attention and multisensory processing in working memory. Consistent with previous work (Busse et al. 2005; Donohue et al. 2011), we show that attention spreads to task‐irrelevant features of an audiovisual compound object, resulting in their automatic encoding into working memory. Critically, this study shows for the first time that the effects of this cross‐modal spread of attention persist throughout the maintenance period of an audiovisual working memory paradigm, albeit electrophysiological results suggest that task‐irrelevant features are not actively maintained. Finally, the observed oscillatory dynamics, particularly in the alpha‐band, show that attentional resources underlying the linking between sensory modalities specifically occur at the retrieval. Further research will be required to conclusively say whether audiovisual working memory storage relies on integrated multisensory object representations.

## Author Contributions

**Ceren Arslan:** conceptualization, formal analysis, investigation, visualization, writing – original draft. **Daniel Schneider:** conceptualization, supervision, writing – review and editing. **Stephan Getzmann:** conceptualization, supervision, writing – review and editing. **Edmund Wascher:** funding acquisition, supervision. **Laura‐Isabelle Klatt:** conceptualization, methodology, project administration, supervision, writing – review and editing.

## Conflicts of Interest

The authors declare no conflicts of interest.

## Supporting information


Data S1.

## Data Availability

The pre‐registration of the sample size rationale, experimental design, data analysis plan, and hypotheses of the study can be found at this link: https://osf.io/c5vjh/.

## Endnotes

^1^
The behavioral data to obtain this a priori effect size measure was kindly provided to us by the authors.
