# Supplementary material for: The Interplay Between Multisensory Processing and Attention in Working Memory: Behavioral and Neural Indices of Audiovisual Object Storage
Source: Psychophysiology. 2025 Feb 21;62(2):e70018. doi: 10.1111/psyp.70018 (PMC11843526; doi:10.1111/psyp.70018)
Supplement: Supplementary file 1 — Data S1. [file PSYP-62-e70018-s001.docx]

**The Interplay Between Multisensory Processing and Attention in Working Memory: Behavioral and Neural Indices of Audio-Visual Object Storage**

Ceren Arslan^1^, Daniel Schneider^1^, Stephan Getzmann^1^, Edmund Wascher^1^, and

Laura-Isabelle Klatt^1^

^1^ Leibniz Research Centre for Working Environment and Human Factors

**Author Note**

Ceren Arslan, arslan@ifado.de [
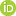
](https://orcid.org/0000-0001-6905-1832) <https://orcid.org/0000-0003-0601-0747>

Daniel Schneider, schneiderd@ifado.de [
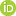
](https://orcid.org/0000-0001-6905-1832) <https://orcid.org/0000-0002-2867-2613>

Stephan Getzmann, getzmann@ifado.de [
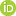
](https://orcid.org/0000-0001-6905-1832) <https://orcid.org/0000-0002-6382-0183>

Edmund Wascher, wascher@ifado.de [
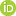
](https://orcid.org/0000-0001-6905-1832) <https://orcid.org/0000-0003-3616-9767>

Laura-Isabelle Klatt, klatt@ifado.de [
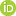
](https://orcid.org/0000-0001-6905-1832) <https://orcid.org/0000-0002-5682-5824>

**Correspondence**

Ceren Arslan, Leibniz Research Centre for Working Environment and Human Factors, Ardeystraße 67, 44139 Dortmund, Germany. E-mail: arslan@ifado.de

**Supplementary Materials**

**1. Artifact Rejection**

We employed a rather conservative criterion for artefact rejection: removing independent components (ICs) with a probability estimate below 50% for the brain category, which resulted in the rejection of 30.68 ICs on average, corresponding to 64% of all ICs (for more details of the preprocessing pipeline, see the ‘EEG Preprocessing’ section in the manuscript). However, excluding 64% of the ICs does not mean excluding 64% of the data. To support our claim, we calculated the percentage of variance represented by individual IC components using the function eeg_pvaf(). Not considering ICs that are labelled as reflecting eye movements, the remaining (rejected) ICs (*M* = 28 ICs, *SD* = 6.71) only accounted for, on average, 11% of the variance of the signal (*SD* = 8.03). Furthermore, Supplementary Figure 1 shows that the current rejection threshold leads to the rejection of artefactual rather than brain-related components of the signal. As the figure shows, two brain-related components at initial positions (IC position at 3 and 4) were rejected for two participants. However, these two brain-related ICs still do not account for a high percentage of variance from overall ICs identified in the data. The rejected component at position 3 explains 3.16% of the variance of the signal, while the IC at position 4 explains 0.70% of the variance. To sum up, here we show that the rejected ICs (not eye movement-related) in the data do not contribute to a high percentage of the variance of the overall signal.


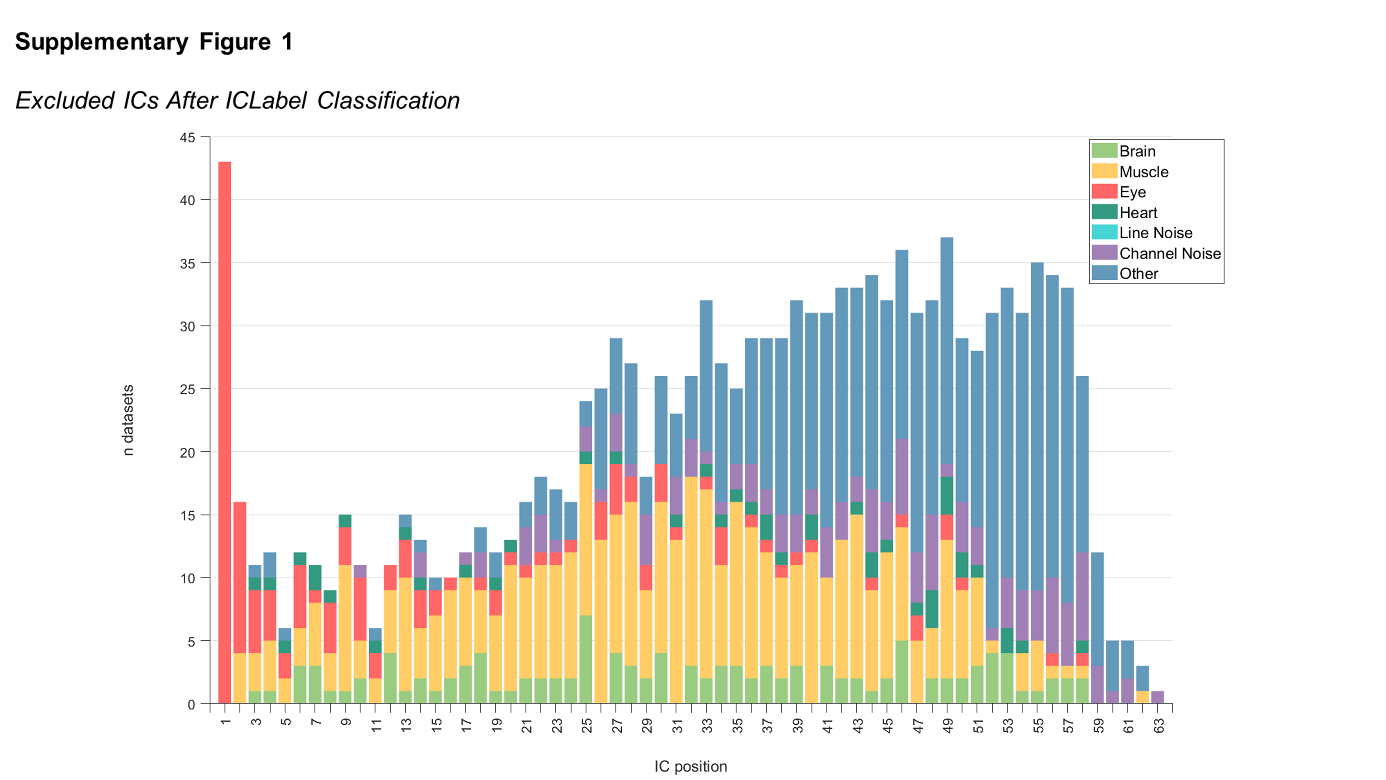


*Note.* The number of excluded ICs is determined by their rank position and color-coded according to the ICLabel category that received the maximum probability estimate. The stacked bar plot can be interpreted as follows: For instance, in 44 datasets (see y-axis), the IC at position 1 was rejected. In all those cases, the respective IC was categorized as belonging to the category “Eye” (that is, it received the highest probability value for the ICLabel category “Eye”). Note that the percent variance accounted for by the back-projection of the respective component decreases as the IC position increases.

**2. Localizer-based channel selection**

***2.1. Localizer task***

A task-independent localizer procedure was employed for the time-frequency analysis to determine channels of interest. This procedure was primarily inspired by the localizer task conducted by van Driel and colleagues (2014) and aimed at specifying electrodes that are maximally sensitive to auditory and visual stimuli processing. Notably, the localizer procedure enabled the selection of regions of interest independent of the experimental manipulations used in the main task. The procedure contained two blocks, an auditory and a visual block, each consisting of 80 trials. Participants were asked to attend to a series of randomly presented atonal tones in the auditory block. The tones were drawn from the same pool of eight sound stimuli presented during the main task, such that each sound was presented ten times. Likewise, in the visual block, participants attended to a series of randomly presented orientations drawn from the pool of stimuli presented during the main task, such that each exposure was repeated ten times. The auditory and visual stimuli were presented for 1000 ms with an inter-stimulus interval of 1000 ms. Participants were not required to respond to any of the stimuli presented. The procedure took approximately six minutes and always followed the main experiment. This task was only completed by a subset of participants (n = 24, 14 females, 2 participants left-handed).

***2.2. Preprocessing***

We followed the preprocessing procedure described in the manuscript in the same steps to pre-process the data from the localizer procedure. Given the shorter duration of each trial, we segmented the data into periods between -700 to 1500 ms relative to the onset of each experimental stimulus. Data were baseline-corrected with the pre-stimulus baseline period of -400 to -100 ms. On average, 3.19 channels were rejected per participant (*SD* = 1.44, range = 1 to 6), and 33.75 components (*SD* = 10.27, range = 16 to 58) were discarded. After pre-processing, on average, 78.29 trials (*SD* = 6.79, 97.86%) remained in the auditory, and 78.92 trials (*SD* = 5, 98.65%) in the visual condition, respectively.

***2.3. Time-frequency decomposition***

To obtain event-related spectral perturbations (ERSPs; Makeig et al., 2004) and inter-trial phase clustering (ITPC, also referred to as phase-locking factor or inter-trial phase coherence; Tallon-Baudry et al., 1996)Morlet wavelet convolution was applied using the same procedure described in the main text. Considering the shorter duration of the trials in the localizer task, the entire pre-stimulus period (i.e., -282 to 0 ms) was chosen as a baseline.

ITPC was estimated to measure the consistency of phase angles over time across trials . As a result of the time-frequency decomposition, one phase angle value was obtained for each time-frequency point and trial. Those phase angles can be mathematically described as vectors with a unit length on a circle (Cohen, 2014). Averaging the phase vectors over trials, ITPC denotes the uniformity of the distribution of phase angles across trials at a given time point. ITPC can range from 0 to 1, reflecting no phase clustering over trials and perfect synchronization. The resulting ITPC time series ranged from -282 to 1082 ms relative to stimulus onset.

Regions of interest were defined by identifying those channels most strongly responding to the auditory and visual experimental stimuli, closely following the approach described by van Driel and colleagues (2014) for a similar purpose. To this end, inter-trial phase clustering activity in the theta (4-8 Hz) and alpha band (8-12 Hz) are considered for the auditory and visual localizer task blocks, respectively (see Supplementary Figure 2A).

***2.4. Cluster-based permutation tests***

The fieldtrip implemented cluster-based permutation tests were performed in the same steps described in the section ‘Cluster-based permutation tests’ in the main text, except that each test was conducted for a time-frequency-channel sampling point instead of a time-frequency pair. A triangulation method defined electrodes as neighbors for contrasts, including the electrode dimension. This method works by calculating a triangulation based on a two-dimensional projection of the channel positions and was used only for the localizer task data. The minimum number of neighboring channels with t-values greater than the observed test statistic to form a cluster was three.

For the auditory condition, ITPC activity within the theta frequency range was contrasted between baseline (-282 to 0 ms relative to stimulus onset) and the post-stimulus activation period (101 to 384 ms relative to stimulus onset), as it was the dominant activity for this condition (see Supplementary Figure 2C). In the visual condition, ITPC increases compared to baseline were evident in both the theta and the alpha-band (see Supplementary Figure 2D). However, we only considered ITPC differences between baseline and activation within the alpha band. This choice was made since the alpha activity is a particularly salient neural oscillation that is maximal in the parieto-occipital regions (Zhigalov et al., 2019; Zhigalov & Jensen, 2020) and for comparability with the approach undertaken by van Driel and colleagues (2014). Data from all electrodes served as input to the cluster-based permutation test. Note that for both contrasts, the resulting significant clusters included all channels. Thus, the topographical distribution of effect sizes was obtained to allow for a meaningful selection of electrodes. Specifically, adjusted partial eta squared (𝜂^2^_p_), defined by Mordkoff (2019), was computed at each electrode. Finally, channels displaying an effect size greater than 1 standard deviation above the mean of effect sizes were selected. This resulted in a slightly right lateralized parieto-occipital cluster of channels for the visual condition (PO4/PO8/PO10, P4, O1/O2/O10/Oz) and one mid-central cluster of channels for the auditory condition (C1/C3/Cz, FC1/FC2/FC3/FC4, F2/F4/Fz). Considering that we did not have any a priori hypotheses about lateralization, we mirrored the channels around the anterior-posterior axis, yielding two channel clusters illustrated in Supplementary Figure 2B.


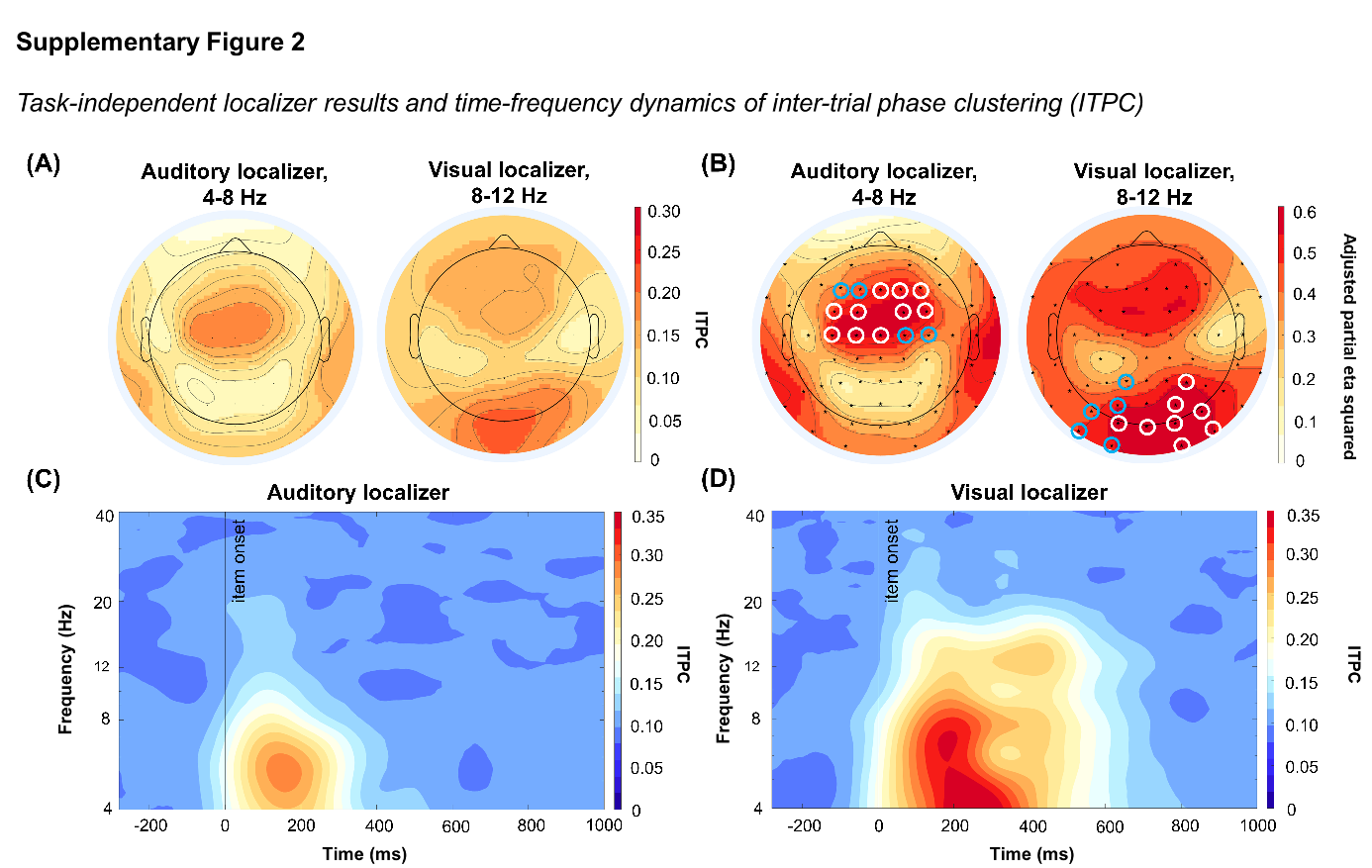


*Note.* (A) Localizer procedure showed a mid-central increase in theta (4-8 Hz) ITPC during the auditory stimuli and parieto-occipital increase in alpha (8-12 Hz) ITPC during visual stimuli presentation (101 to 384 ms post-stimulus onset); (B) Electrodes of interest were selected based on the topographical distribution of effect sizes. Electrodes highlighted with white discs indicate channels exceeding the pre-defined threshold of 1 SD above the mean. Electrodes with blue discs were additionally selected to create symmetric electrode clusters; (C) Time-frequency dynamics of ITPC for the auditory stimuli showed enhanced synchronization in the theta band; and (D) Time-frequency dynamics of ITPC for the visual stimuli showed enhanced synchronization in the theta and alpha band.

**3. Is the probe congruency effect affected by the task-relevance of a mis-matching feature?**

To explore whether the probe congruency effect is modulated by the task relevance of the interference-inducing feature of the probe, separate sets of rmANOVAs were conducted, contrasting the conjunction condition with the single-feature conditions. Critically, only trials requiring a ‘no’ response were included for these contrasts because ‘yes’ responses are typically faster than ‘no’ responses (Brouillet et al., 2010). This accounts for the fact that ‘yes’ and ‘no’ responses are not equally distributed across congruent and incongruent trials in the conjunction condition (50% of the conjunction trials required a congruent ‘yes’ response, while 1/3 of the remaining 50% required congruent and incongruent ‘no’ responses; for more details, see Figure 2 in the manuscript). This manipulation in the conjunction condition was done to ensure an overall equal number of ‘yes’ and ‘no’ responses in this condition.

A group of rmANOVAs was run with factors memory conditions (auditory vs. conjunction) and set size (1 vs. 2) on the RT and accuracy differences between congruent (visual feature no-match + auditory feature no-match) vs. incongruent (visual feature match + auditory feature no-match) probe trials served as dependent variables. A significant main effect of memory condition on the accuracy, *F*(1, 43) = 36.51, *p* < .001, 𝜂^2^_p_ = 0.46, BF_10_ > 1000, showed a greater probe congruency effect for the conjunction condition (*M* = 14.89, *SD* = 13.83) compared to the auditory condition (*M* = 2.69, *SD* = 7.44) (see Supplementary Figure 3A). This suggests greater interference results from a task-relevant but non-matching feature than a task-irrelevant incongruent feature. Planned contrasts verified the congruency effect both in the auditory, *t*(43) = 2.40, *p* = .02, *d* = 0.36, BF_10_ = 2.11, and in the conjunction conditions, *t*(43) = 7.14, *p* < .001, *d* = 1.08, BF_10_ >1000. Additionally, there was a significant main effect of the set size, *F*(1, 43) = 24.26, *p* < .001, 𝜂^2^_p_ = 0.09, BF_10_ = 578.94, indicating a stronger probe congruency effect for a higher memory load (see Supplementary Figure 3A). Yet, the interaction of the two factors was not significant, *F*(1, 43) = 1.05, *p* = .31, 𝜂^2^_p_ = 0.003, BF_10_ = 0.33.

Similar to the accuracy results, a significant main effect of condition in RTs, *F*(1, 43) = 7.21, *p* =.01, 𝜂^2^_p_ = 0.07, BF_10_ = 4.56, indicated a greater probe congruency effect for the conjunction condition (*M* = 74.21, *SD* = 138.83) compared to the auditory condition (*M* = 16.90, *SD* = 82.97) (see Supplementary Figure 3C). Planned follow-up contrasts established the congruency effect in the conjunction condition, reflecting slower RTs in the congruent compared to incongruent trials, *t*(43) = 3.55, *p* < .001, *d* = 0.54, BF_10_ = 30.78, but not in the auditory condition, *t*(43) = 1.35, *p* = .18, *d* = 0.20, BF_10_ = 0.38. Further, there was neither a significant interaction of condition and set size, *F*(1, 43) = 0.06, *p* = .81, 𝜂^2^_p_ = 0.25, BF_10_ = 0.23, nor a main effect of set size, *F*(1, 43) = 1.53, *p* = .22, 𝜂^2^_p_ = 0.01, BF_10_ = 0.40.

A second group of rmANOVAs was performed, including the factors memory condition (visual vs. conjunction) and set size (1 vs. 2) on the RT and accuracy differences between congruent (visual feature no-match + auditory feature no-match) and incongruent (visual feature no-match + auditory feature match) probe trials. Accuracies for memory conditions significantly differed from each other, *F*(1, 43) = 6.82, *p* = .012, 𝜂^2^_p_ = 0.06, BF_10_ = 3.72, indicating a greater probe congruency effect for the conjunction condition (*M* = 5.34, *SD* = 6.64), compared to the visual condition (*M* = 1.63, *SD* = 5.98) (see Supplementary Figure 3B). This result suggests that greater interference results from a task-relevant but non-matching feature, leading to lower accuracy than a task-irrelevant, incongruent feature. Planned follow-up contrasts verified the presence of an interference effect (i.e., congruency effect) in the conjunction condition, *t*(43) = 5.34, *p* < .001, *d* = 0.81, BF_10_ > 1000, reflecting better performance for congruent compared to incongruent trials, but not in the visual condition, *t*(43) = 1.81, *p* = .08, *d* = 0.27, BF_10_ = 0.73. Neither the main effect of set size, *F*(1, 43) = 2.53, *p* =.12, 𝜂^2^_p_ = 0.06, BF_10_ = 0.59, nor the interaction of condition and set size in accuracies, *F*(1, 43) = 0.18, *p* =.68, 𝜂^2^_p_ = 0.004, BF_10_ = 0.24, was significant. The RT analysis yielded no significant main effect of condition (*F*(1, 43) = 0.11, *p* = .74, 𝜂^2^_p_ = 0.003, BF_10_ = 0.23) or of set size (*F*(1, 43) = 0.18, *p* = .68, 𝜂^2^_p_ = 0.004, BF_10_ = 0.23) nor an interaction (*F*(1, 43) = 0.05, *p* = .83, 𝜂^2^_p_ = 0.001, BF_10_ = 0.01) (see Supplementary Figure 3D).

Overall, for accuracy, we consistently find that interference in incongruent probe trials is greater if participants attend both modalities. Congruency effects in terms of RTs were only apparent when considering ‘no’ response trials in the conjunction condition in which the auditory feature was task-relevant; here, faster responses were evident for congruent compared to incongruent trials. Furthermore, we see that the probe congruency effect in the single-feature conditions, only including the ‘no’ response trials, is not as strong as when both ‘yes’ and ‘no’ responses are included (for the latter result, see Figure 3 on page 15).


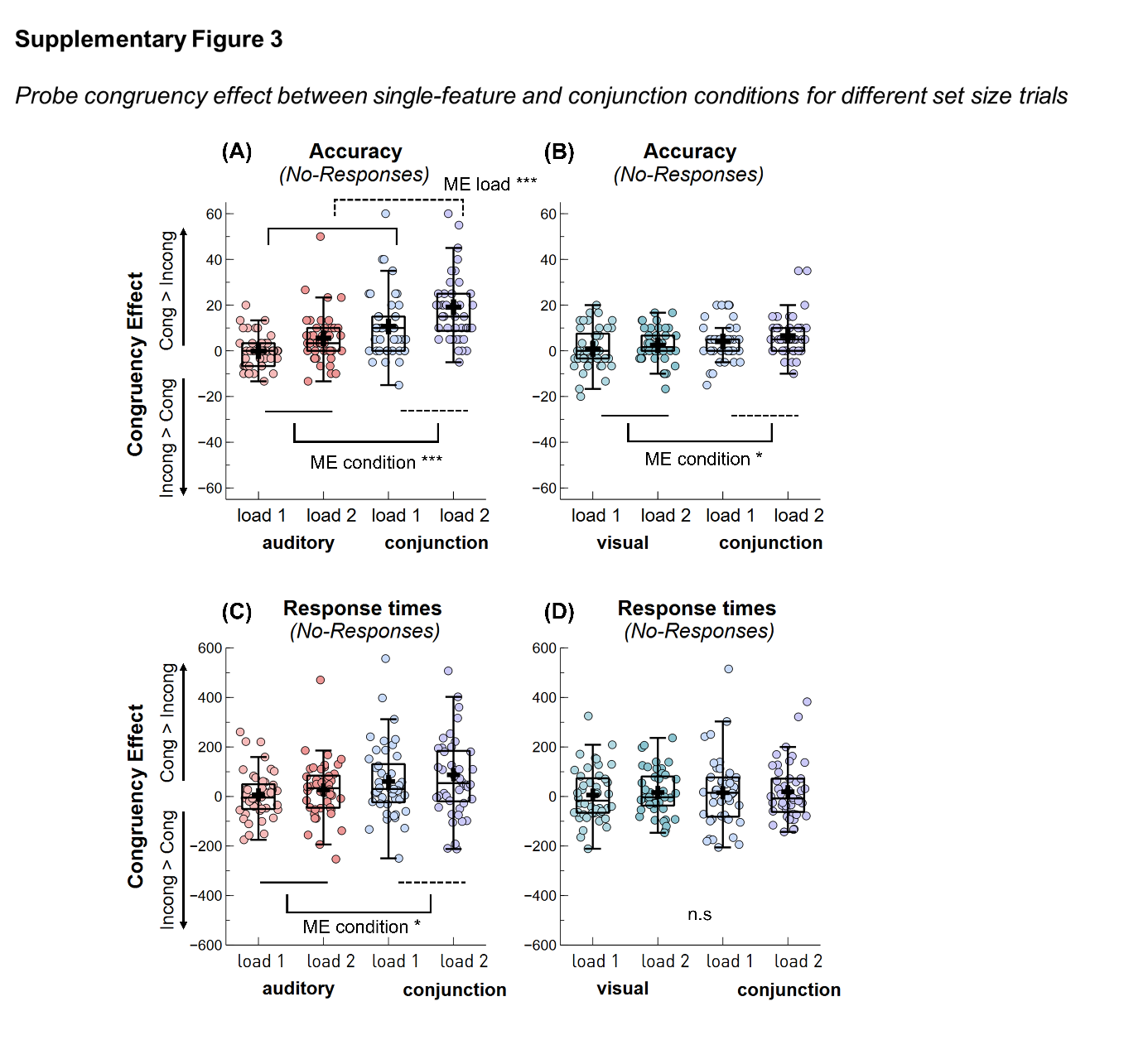


*Note.* (A) Proportion of correct responses for the congruency effect between auditory and conjunction conditions, collapsed over trials requiring a ‘no’ response. The data depicted for the conjunction condition refers to trials with a probe containing an auditory-no-match feature paired with a visual-match feature; (B) Proportion of correct responses for the congruency effect between visual and conjunction conditions, averaged over trials requiring a ‘no’ response. The data depicted for the conjunction condition refers to trials with a probe containing a visual-no-match feature paired with an auditory-match feature; (C) RTs for the congruency effect between auditory and conjunction conditions, collapsed over trials requiring a ‘no’ response. The data depicted for the conjunction condition refers to trials with a probe containing an auditory-no-match feature paired with a visual-match feature; (D) RTs for the congruency effect between visual and conjunction conditions, averaged over trials requiring a ‘no’ response. The data depicted for the conjunction condition refers to trials with a probe containing a visual-no-match feature paired with an auditory-match feature. Boxplots show the +/- 1.5 interquartile range and the median. The dots illustrate individual participant averages per condition. A black cross illustrates the condition mean.

**p* <.05, ****p* < .001, n.s = not significant.

A closer observation of the data in comparison to the results reported in the main manuscript (including both ‘yes’ and ‘no’ responses) reveals that the probe congruency effect appears diminished (or even disappears) for some of the single-feature conditions in particular, in response times. This suggests that interference from a task-relevant feature may be particularly strong in ‘yes’ response trials.

Unsurprisingly, ‘yes’ and ‘no’ response trials are qualitatively different. In fact, it is commonly observed that responses in ‘yes’ response trials are faster than in ‘no’ response trials (as they confirm rather than refute a match with memory; see e.g., Brouillet et al., 2010). Accordingly, in ‘yes’ response trials, when both auditory and visual features are congruent with the memory sample, participants likely benefit from an automatic match detection process, which enhances response speed and accuracy. This is also confirmed in our data, in which congruent ‘yes’ responses are more accurate (M_acc_ = 94%) and faster (M_RT_ = 881 ms) than congruent ‘no’ responses (M_acc_ = 88.95%, M_RT_ = 820 ms). Hence, in ‘yes’ response trials, the congruency effect might be especially pronounced because it may take more active cognitive control to resolve the conflict between the strong “yes” signal (amplified through initial automatic-template matching of the attended feature) and the arising interference from the task-irrelevant feature (signalling a ‘no’ response) in partial-repetition (i.e., incongruent) trials. In ‘no’ response trials, this interference may be less strong, given that mismatch detection (i.e., an initial no-match with working memory contents of the attended modality) engages controlled and deliberate processing to confirm the absence of a match.

**4.Oscillatory activity in memory conditions**

Supplementary Figure 4 shows the time course of oscillatory power for all three memory conditions for anterior (Figure 4A) and posterior electrode clusters (Figure 4B), collapsed across trials with different set sizes. Inspecting the time course of oscillatory power, a similar pattern emerges across all three memory conditions: In response to the item and mask presentations, a stimulus-evoked increase in the theta band and a decreased alpha and beta band power is evident. Shortly after encoding and in the delay interval, this activity was followed by an increase in power in the upper alpha and lower beta bands. After the probe presentation, a prominent decrease in alpha- and lower beta power and an increase in theta power can be observed.

**
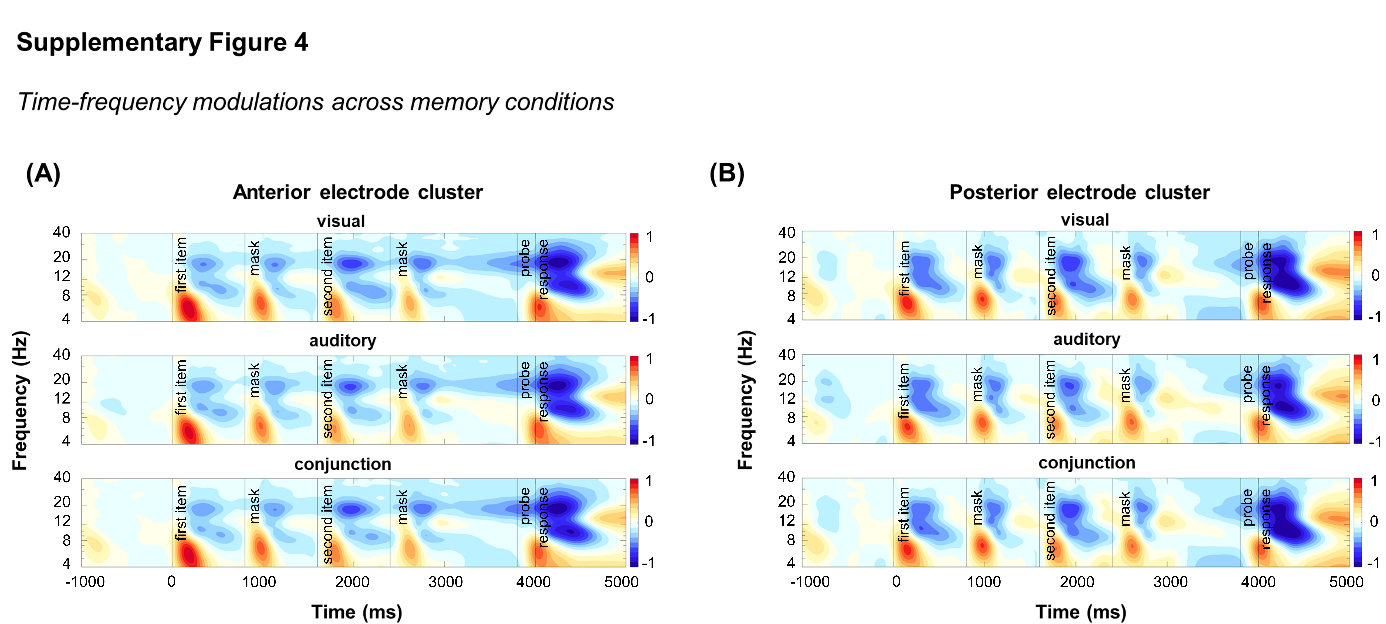
**

*Note.* Power was averaged across (A) the midcentral electrode cluster, including C1/C2/C3/C4/Cz, FC1/FC2/FC3/FC4, F1/F2/F3/F4/Fz; and (B) the parieto-occipital electrode cluster including PO3/PO4/PO7/PO8/ PO9/PO10, P3/P4, O1/O2/O9/O10/Oz for each condition.

**5. Follow-up Questionnaire**

We asked follow-up questions to evaluate whether participants followed the task instructions to ignore the task-irrelevant features such as 1) "In the auditory task, were you able to focus only on the sounds, and did you try to ignore the visual stimuli?", 2) "In the visual task, were you able to focus only on the visual stimuli, and did you try to ignore the sounds?". Participants rated their answers on a scale from 1 (never) to 5 (always). Overall, the answers to the self-report questionnaire show that participants almost always attended to the task-relevant features while ignoring task-irrelevant features. Supplementary Table 1 shows the mean and standard deviation of the ratings to those questions for attend-auditory and attend-visual conditions.


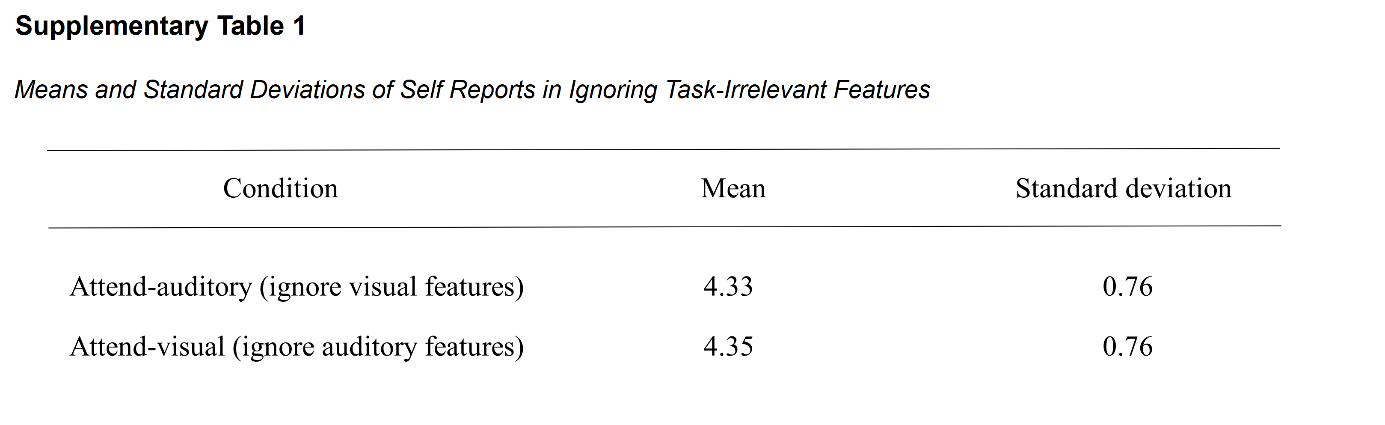


*Note.* Means and standard deviations of the answers to the task-instruction-related follow-up questions on a scale from 1 (never) to 5 (always).

**References**

Brouillet, T., Heurley, L., Martin, S., & Brouillet, D. (2010). The embodied cognition theory and the motor component of “yes” and “no” verbal responses. *Acta Psychologica*, *134*(3), 310–317. https://doi.org/10.1016/j.actpsy.2010.03.003

Cohen, M. X. (2014). *Analyzing Neural Time Series Data: Theory and Practice*. The MIT Press. https://doi.org/10.7551/mitpress/9609.001.0001

Delorme, A., & Makeig, S. (2004). EEGLAB: An open source toolbox for analysis of single-trial EEG dynamics including independent component analysis. *Journal of Neuroscience Methods*, *134*(1), 9–21. https://doi.org/10.1016/j.jneumeth.2003.10.009

Makeig, S., Debener, S., Onton, J., & Delorme, A. (2004). Mining event-related brain dynamics. *Trends in Cognitive Sciences*, *8*(5), 204–210. https://doi.org/10.1016/j.tics.2004.03.008

Mordkoff, J. T. (2019). A Simple Method for Removing Bias From a Popular Measure of Standardized Effect Size: Adjusted Partial Eta Squared. *Advances in Methods and Practices in Psychological Science*, *2*(3), 228–232. https://doi.org/10.1177/2515245919855053

Tallon-Baudry, C., Bertrand, O., Delpuech, C., & Pernier, J. (1996). Stimulus Specificity of Phase-Locked and Non-Phase-Locked 40 Hz Visual Responses in Human. *The Journal of Neuroscience*, *16*(13), 4240–4249. https://doi.org/10.1523/JNEUROSCI.16-13-04240.1996

van Driel, J., Knapen, T., van Es, D. M., & Cohen, M. X. (2014). Interregional alpha-band synchrony supports temporal cross-modal integration. *NeuroImage*, *101*, 404–415. https://doi.org/10.1016/j.neuroimage.2014.07.022

Zhigalov, A., Herring, J. D., Herpers, J., Bergmann, T. O., & Jensen, O. (2019). Probing cortical excitability using rapid frequency tagging. *NeuroImage*, *195*, 59–66. https://doi.org/10.1016/j.neuroimage.2019.03.056

Zhigalov, A., & Jensen, O. (2020). Alpha oscillations do not implement gain control in early visual cortex but rather gating in parieto‐occipital regions. *Human Brain Mapping*, *41*(18), 5176–5186. https://doi.org/10.1002/hbm.25183
